# Progress in Antimelanoma Research of Natural Triterpenoids and Their Derivatives: Mechanisms of Action, Bioavailability Enhancement and Structure Modifications

**DOI:** 10.3390/molecules28237763

**Published:** 2023-11-24

**Authors:** Marta Grudzińska, Bogna Stachnik, Agnieszka Galanty, Agnieszka Sołtys, Irma Podolak

**Affiliations:** 1 Department of Pharmacognosy, Jagiellonian University Medical College, Medyczna 9, 30-688 Kraków, Poland; marta.grudzinska@doctoral.uj.edu.pl (M.G.); bogna.stachnik@gmail.com (B.S.); agnieszka.soltys@doctoral.uj.edu.pl (A.S.); irma.podolak@uj.edu.pl (I.P.); 2 Department of Food Chemistry and Nutrition, Jagiellonian University Medical College, Medyczna 9, 30-688 Kraków, Poland; 3 Doctoral School of Medical and Health Sciences, Jagiellonian University Medical College, Łazarza 16, 31-530 Kraków, Poland

**Keywords:** triterpenoids, antimelanoma activity, delivery systems, structural modifications

## Abstract

Melanoma is one of the most dangerous forms of skin cancer, characterized by early metastasis and rapid development. In search for effective treatment options, much attention is given to triterpenoids of plant origin, which are considered promising drug candidates due to their well described anticancer properties and relatively low toxicity. This paper comprehensively summarizes the antimelanoma potential of natural triterpenoids, that are also used as scaffolds for the development of more effective derivatives. These include betulin, betulinic acid, ursolic acid, maslinic acid, oleanolic acid, celastrol and lupeol. Some lesser-known triterpenoids that deserve attention in this context are 22*β*-hydroxytingenone, cucurbitacins, geoditin A and ganoderic acids. Recently described mechanisms of action are presented, together with the results of preclinical in vitro and in vivo studies, as well as the use of drug delivery systems and pharmaceutical technologies to improve the bioavailability of triterpenoids. This paper also reviews the most promising structural modifications, based on structure–activity observations. In conclusion, triterpenoids of plant origin and some of their semi-synthetic derivatives exert significant cytotoxic, antiproliferative and chemopreventive effects that can be beneficial for melanoma treatment. Recent data indicate that their poor solubility in water, and thus low bioavailability, can be overcome by complexing with cyclodextrins, or the use of nanoparticles and ethosomes, thus making these compounds promising antimelanoma drug candidates for further development.

## 1. Introduction

Melanoma is a form of skin cancer, characterized by rapid development, high mortality and poor prognosis [1,2]. It is the most dangerous type of skin cancer due to its prominent metastasis and resistance to radio- and chemotherapy [3]. Changes in lifestyle, including exposure to excessive UV radiation, have led to a significant increase in the amount of melanoma incidence and melanoma-related deaths over the years; this accounts for 75% of skin cancer deaths [4,5]. Nowadays, standard melanoma treatment is mainly based on surgical procedures (at early stages), chemotherapy, radiation, immunotherapy and targeted therapies; nevertheless, effective treatment options in melanoma are still limited [3]. Multi-drug resistance and severe side effects are the main drawbacks of chemotherapeutic agents used in skin cancer [4,6]. Thus, new therapies are still sought, and nature is considered to be a vast source of bioactive compounds with a potential antimelanoma effect.

Natural compounds of plant origin play an important role in drug discovery and development. Today, the majority of anticancer and anti-infectious agents are based on natural compounds [7]. Triterpenoids are one of the classes of potentially active compounds, widely distributed in plants [3]. They are known for their anti-inflammatory, analgesic, bactericidal, virostatic and hepatoprotective activity [8]. Moreover, many studies have confirmed their cytotoxic properties in vitro against various types of cancer [4], due to modulating the pathways that are involved in cell proliferation and apoptosis [9]. Some recent reviews on the general anticancer activity of triterpenoids have focused on the development of semi-synthetic derivatives [10,11,12] or on the efficacy of the most widely studied compound, betulinic acid (BA) [13,14,15,16,17], and also oleanolic acid (OA) [18]. In 2022, a review paper describing the anticancer activity of various terpenoids, including triterpenoids such as BA, ursolic acid (UA) and lupeol (LU), was published [19]. The only review, in which more emphasis was put on the treatment of melanoma, was devoted solely to betulin (BT) [20].

Taking into account the need for new drug candidates that could combat this most dangerous type of skin cancer, in this paper we provide an overview of current trends and achievements in research conducted on triterpenoids, with respect to their antimelanoma potential. In vitro and in vivo data against human and murine melanoma cell lines are included, and the most promising compounds are discussed. In addition, the efforts to increase triterpenoid bioavailability are presented, together with the mechanisms of cytotoxic activity. Finally, modifications based on structure–activity relationships are summarized.

The review covers the studies published from 2010 to 2022. The following databases were searched: MEDLINE/PubMed, SCOPUS/Elsevier, Springer/ICM, Google Scholar and the key words used were natural triterpenoids, triterpenoids, antimelanoma activity and anticancer. We found around 600 records from which we selected articles that addressed the antimelanoma activity of natural triterpenoids and their derivatives were tested both in vitro and in vivo. As several previously published reviews concern the cytotoxic activity of triterpenoid saponins [21,22,23,24], we have limited our search only to free triterpenoids; we also excluded review articles and research focusing on the activity of extracts instead of pure compounds.

## 2. Natural Triterpenoids

Triterpenoids are widely distributed natural metabolites, found predominantly in plants and fungi. Formed via the mevalonate pathway, they are biosynthesized in a wide array of structural types, of which the most common and generally bioactive are cyclic forms. Pentacyclic triterpenoids are characterized by a 30-carbon backbone with four six-membered rings and one five-membered ring (hopanes and lupanes) or five six-membered rings (oleananes, lanostanes and ursanes). Often studied and found in the bark of various birch species, pentacyclic triterpenoid is BT [25]. As mentioned earlier, the cytotoxic activity of pentacyclic triterpenoids has been widely reported.

Another group of cyclic triterpenoids that exhibit a broad spectrum of anticancer properties are tetracyclic structures, such as cucurbitacins or ganoderic acids. Cucurbitacins are highly oxygenated tetracyclic triterpenoids, isolated from the plants of the Cucurbitaceae family, e.g., pumpkin, melons, squash. Nineteen different cucurbitacins (Cucs) have been described so far. Among them, Cucs E, B, D and Q showed a promising antiproliferative activity on various cancer cell lines. Additionally, Cuc B was reported to act as a hepatoprotective agent, whereas Cuc R revealed anti-inflammatory activity [26,27,28]. Structural analysis of these triterpenoids shows that all of them contain certain moieties, such as C-2 hydroxy group, a double bond at the C-5 and/or C-1 positions, C-25 hydroxy or acetoxy group, a ketone group at the C-3 and C-11 positions and 16*α*,20*β*-dihydroxy groups, that could be important for cytotoxicity against cancer cells [11]. Ganoderic acids are fungal metabolites, they are typical of *Ganoderma lucidum*, a mushroom species, that has been used for centuries in the Far East, not only to improve health and promote longevity, but also in the treatment of many diseases, including cancer [29].

## 3. Antimelanoma Activity of Natural Triterpenoids—Evidence from In Vitro Studies

The data on the cytotoxic activity of natural triterpenoids on different melanoma cell lines are gathered in Table 1 and Table 2. It is interesting to note that, as opposed to studies on other types of cancer, in the case of melanoma, quite a significant number of experimental data on triterpenoids are derived from tests performed on murine cell lines. Studies on human melanoma cell lines have only a slight predominance. Surprisingly, for some compounds, such as BA, the human cell lines seem to be more sensitive than the animal ones. Furthermore, only a small number of experiments provided data on the selectivity of the tested compounds, by studying their concomitant impact on normal cell lines [30,31], and even fewer compared the results with the activity of a reference cytostatic drug [9,30,32].

Nevertheless, it is worth pointing out that in the majority of these studies, triterpenoids were less toxic to normal cells, in comparison to cancer cells. The most prominent example is maslinic acid, as the concentrations at which it affected normal cells were even 20 times higher than those needed for the induction of apoptosis in cancer cells. The cytotoxic doses of the reviewed triterpenoids varied from a few to almost 300 μM, depending on the type of cell line and compound structure.

Data covered in this review clearly show that BT and BA were more potent than LU, UA or OA, which seldom reached IC_50_ below 10.0 μM. An interesting observation was made in the experiment on four human melanoma cell lines: A375, MM200, Mel-RM, Me4405 and human fibroblasts HFFF2, treated with UA and cisplatin, were used as a reference drug. UA was more potent towards melanoma cells, for example, the Me4405 line after 24 h of incubation, with IC_50_ 28.67 ± 0.05, as compared to cisplatin IC_50_ 180.3 ± 15. In the case of healthy HFFF2 cells, UA was less active (46.71–69.77 μM after 48 and 24 h) compared to cancer cells used in this experiment, which indicates selectivity of action. The exception was the MM200 cells, for which the activity was comparable (33.09–61.6 μM after 48 and 24 h) [30]. Interesting results regarding the activity of UA were obtained in one of the studies against B16F1 cells. UA was shown to induce melanosome autophagy and inhibit the action of α-melanocyte-stimulating hormone (*α*-MSH). Based on these results, the researchers concluded that UA, by increasing the degradation of melanosomes in melanocytes of B16F1 cells, inhibits skin pigmentation [33]. In one of the studies [34], it was shown for the first time that BA in combination with taxanes leads to a reduction in the viability of melanoma cells (FM55P, FM55M2, A375, SK-MEL-28). This compound in combination with docetaxel or paclitaxel induced a synergistic effect and a better response to treatment in vitro. Interesting effects were also observed in a study in which BA was combined with digoxin and their effect on SK-Mel-28 and RPMI-7951 cells was investigated. The best results were obtained by combining BA in a dose of 10 μM with digoxin in a dose of 50 nM, which resulted in a decrease in cell viability to 17% and 23% for SK-MEL-28 and RPMI-7951 cells, respectively. In comparison, 10 μM of BA alone reduced cell viability to approximately 50% and 88% for SK-MEL-28 and RPMI-7951 cells and 50 nM of digoxin alone reduced cell viability to 80% and 82% for SK-MEL-28 and RPMI-7951 [35].

Another pentacyclic compound that is worth mentioning is ilexgenin A, isolated from *Ilex hainanensis*. The compound inhibited the viability of B16-F10 cells in a dose- and time-dependent manner, with IC_50_ values of 27.34 and 12.44 μM after 24 and 48 h, respectively. As was shown by flow cytometry, the compound induced dose-dependent growth arrest in the G1-S phase of the cell cycle in B16-F10 cells. Moreover, ilexgenin A significantly inhibited the IL-6 (interleukin-6) production in macrophages stimulated by MCM (melanoma conditioned medium) [36].

**Table 1:** Cytotoxic in vitro activity of natural triterpenoids against melanoma cell lines.

Compound	Cell Line	Results	References
Betulin (BT)	SK-MEL-28^H^	IC_50_ 16.2 μM (48 h)	[1]
	SK-MEL-2^H^	IC_50_ > 45.2 μM (48 h)	
		IC_50_ 1.1–4.6 μg/mL (72 h)	[37,38]
		IC_50_ > 250 μM (48 h)	[1]
		IC_50_ 4.1 μM	[9]
	Me-45^H^	IC_50_ 24.2–30.5 μM (24–72 h)	[3]
	G361^H^	IC_50_ 12.4 μM (48 h)	[1]
	Hs294T^H^	IC_50_ 44.1 μM	[39]
	A-431^H^	Inhibition of proliferation: ≈ 68% of control (C = no data)	[40]
		IC_50_ 6.8 μM (72 h)	[41]
		Inhibition of proliferation: 52–62% of control (C = no data)	[42]
	B164A5^A^	IC_50_ 4.27 μM (72 h)	[43]
	B16Ova^A^	IC_50_ 3.89 μM (72 h)	
	B16-2F2^A^	IC_50_ 27.4 μM (48 h)	[1]
	B16-F10^A^	IC_50_ 13.8 μM (48 h)	
		IC_50_ 14.38 μM (48 h)	
	C-32^H^	IC_50_ 15.61 μM (72 h)	[44]
Betulinic acid (BA)	MEL-1^H^	ED_50_ 3.3 μg/mL (72 h)	[45]
	MEL-2^H^	IC_50_ 1.3 μM	[46]
		ED_50_ 1 μg/mL (72 h)	[45]
		ED_50_ 1.2 μg/mL (72 h)	[47]
	G361^H^	IC_50_ 5.2 μM (48 h)	[1]
	SK-MEL-28^H^	IC_50_ 6.5 μM (48 h)	
		IC_50_ 2.21 μM (72 h)	[34]
		Cell viability ~50% (C = 10 μM) (24 h)	[35]
	RPMI-7951^H^	Cell viability: ~88% (C = 10 μM) (24 h)	
	518A2^H^	IC_50_ 8.13 μM (96 h)	[48]
	Me-45^H^	IC_50_ 22.7–15.3 μM (24–72 h)	[3]
	MEL-1,-3,-4^H^	IC_50_ 1.1–4.6 μg/mL (72 h)	[37]
	MEL-1,-2,-4^H^	IC_50_ 0.5–4.8 μg/mL (72 h)	
	B16-2F2^A^	IC_50_ 7.9 μM (48 h)	[1]
	B16^A^	IC_50_ 76 μg/mL (48 h)	[49]
	B16-F1^A^	IC_50_ 16.1 μM	[46]
	B16-F10^A^	IC_50_ 16.41 μM (48h	[1]
		IC_50_ 70 μM	[50]
	B164A5 metastatic^A^	Cell viability: 57.89% (C = no data)	[51]
	B164A5 non-metastatic^A^	Cell viability: 61.82%	
	WM-266-4^H^	Inhibition of proliferation: < 20% of control (C > 2 μM)	[52]
	A375^H^	IC_50_ 16.91 µM (24 h)	[53]
		IC_50_ 154 μM	[4]
		IC_50_ 15.94 μM (72 h)	[34]
		Cell viability: 75% (C = 50 µM) (24 h)	[54]
Betulinic acid (BA)	eRGO1^A^	IC_50_ 22.8 µM/L (24 h)	[55]
		IC_50_ 20.7 µM/L (48 h)	
		IC_50_ 12.7 µM/L (96 h)	
	MelDuWi^A^	IC_50_ 34.6 µM/L (24 h)	
		IC_50_ 31.7 µM/L (48 h)	
		IC_50_ 23.6 µM/L (96 h)	
	FM55P^H^	IC_50_ 5.62 μM (72 h)	[34]
	FM55M2^H^	IC_50_ 4.08 μM (72 h)	
Lupeol (LU)	WM35^H^	IC_50_ 32 µM/L (72 h)	[56]
	451Lu^H^	IC_50_ 38 µM/L (72 h)	
	G361^H^	Cell growth inhibition: 97.5% (C = 10 µM) (72 h)	[57]
		IC_50_ 50 µM (72 h)	[58]
	Mel 928^H^	IC_50_ 75 µM (48 h)	[59]
	Mel 1241^H^	IC_50_ 72 µM (48 h)	
	Mel 1011^H^	IC_50_ 135 µM (48 h)	
	B16F10^A^	IC_50_ 58.39 µg/mL (24 h)	[32]
	A375^H^	IC_50_ 66.59 µM (24 h)	[60]
	RPMI-7951^H^	IC_50_ 45.54 µM (24 h)	
Oleanolic acid (OA)	A2058^H^	IC_50_ 60 µM (48 h)	[61]
	A375^H^	IC_50_ 75 µM (48 h)	
		Cell viability: 74.8% (C = 50 µM) (24 h)	[54]
	WM-266-4^H^	Inhibition of proliferation: 21% of control (C = 20 μM) (24 h)	[52]
	A375SM^H^	Cell viability: 79.6% (C = 80 µM)	[62]
		Cell viability: 41.5% (C = 100 µM)	
	A375P^H^	Cell viability: 82.4% (C = 60 µM)	
		Cell viability: 46.4% (C = 100 µM)	
Ursolic acid (UA)	A375^H^	IC_50_ 75 μM	[30]
		Cell viability: 85% (C = 50 µM) (24 h)	[54]
		IC_50_ 68.22 μM (48 h)	[63]
		GI_50_ 26 μM	[64]
		IC_50_ 6.95 µg/mL (24 h)	[65]
		IC_50_ 5.20 µg/mL (48 h)	
		IC_50_ 32.36 μM (24 h)	[26]
		IC_50_ 19.45 μM (48 h)	
	SK-MEL 2^H^	IC_50_ 58.43 μM (48 h)	[66]
		IC_50_ 58.44 μM (48 h)	[63]
	A2058^H^	IC_50_ 60 μM (48 h)	[61]
	B164A5^A^	IC_50_ 43.59 μM (48 h)	[63]
	B16F10^A^	IC_50_ 31.65 μM (24 h)	[67]
	Mel-RM^H^	IC_50_ 26.25 μM (48 h)	[30]
		IC_50_ 40.48 μM (24 h)	
	Me4405^H^	IC_50_ 28.67 μM (24 h)	
		IC_50_ 18.15 μM (48 h)	
Ursolic acid (UA)	MM200^H^	IC_50_ 33.09 μM (48 h)	[30]
		IC_50_ 61.6 μM (24 h)	
	HFFF2^H^	IC_50_ 46.71 μM (48 h)	
		IC_50_ 69.77 μM (24 h)	
	HTB-140^H^	IC_50_ 5.69 µg/mL (24 h)	[65]
		IC_50_ 4.13 µg/mL (48 h)	
	WM793^H^	IC_50_ 5.89 µg/mL (24 h)	
		IC_50_ 4.08 µg/mL (48 h)	
	WM-266-4^H^	Inhibition of proliferation: 10% of control (C = 10 μM) (24 h)	[52]
Ursolic acid + oleanolic acid (ratio 1:1 and 3.5:1)	WM-266-4^H^	Inhibition of proliferation: 20% (4 h), 7% (24 h), 6% (48 h) of control (C = 10 μM)	[52]
Ursolic acid + oleanolic acid (ratio 1:1)	A375^H^	IC_50_ 60 µM (48 h)	[61]
	A2058^H^		

The letters in superscript at the cell line name mean A—animal cell line, H—human cell line. Cell lines: G-361 human Caucasian malignant melanoma; Hs294T human melanoma; SK-MEL-1,-2,-3,-4 human malignant melanoma; SK-MEL-28 human malignant melanoma; Me-45 human malignant melanoma; A-431 human squamous carcinoma; B164A5 mouse melanoma; B16Ova murine melanoma transfected with an ovalbumin; B16F10, B16F1, B162F2, B164A5 murine melanoma; 518A2 human melanoma; C-32 human amelanotic melanoma; A375 human malignant melanoma; A375SM, A375P human melanoma; WM35 human primary melanoma; 451Lu human metastasis melanoma; Mel 928, Mel 1241, Mel 1011, Me4405, MM200 human melanoma; HFFF2 human Caucasian foetal foreskin fibroblast; Mel-RM, Me45 human melanoma from metastatic site; WM-266-4 human skin metastatic melanoma; RPMI-7951 human skin malignant melanoma; HTB-140 human melanoma derived from metastatic site; WM793 human stage I primary melanoma; FM55P human primary malignant melanoma; FM55M2 human metastases malignant melanoma; eRGO1, MelDuWi primary equine melanoma. Abbreviations: C—concentration; IC_50_—median inhibitory concentration; ED_50_—median effective dose; GI50—median growth inhibition.

Taraxasterol is a pentacyclic triterpenoid compound found in dandelion (*Taraxacum officinale*). In the study by Liu et al. [68], it was shown that this compound at concentrations above 10 μg/mL significantly inhibited SK-MEL-28 and A375 cell viability, and induced apoptosis by increasing the number of apoptotic cells and the expression of PARP and caspase-3 proteins in comparison to control. In addition, an increase in the amount of intracellular ROS, inhibition of migration and invasiveness of melanoma cells was found after application of the compound.

Among the tested tetracyclic triterpenoids, the lowest IC_50_ values were obtained for natural cucurbitacins [26] and celastrol (CEL) [69]. Cucurbitacins can be regarded as promising antimelanoma compounds, with reported IC_50_ values usually lower than 2 μM (Table 2). Cucs E, B and D exhibited a cytotoxic effect on the SK-MEL-28 and A375 cell lines, with IC_50_ values 0.36 ± 0.14 and 1.54 ± 0.04 μM, respectively [26].

Another promising antimelanoma drug candidate among natural triterpenoids of marine origin is geoditin A. This compound was isolated from various marine sponges, such as *Geodia japonica*, native to the South China Sea. The compound belongs to a rare three-ring group of triterpenoids of isomalabaricane type. In recent years, geoditin A has received special attention as it inhibited tumour cell proliferation via cyclin-dependent kinase activity mechanism, making it a promising candidate for further development as a chemotherapeutic drug. Geoditin A is also an oxidative stressor, which may interfere with the melanogenesis process and tyrosinase activity in melanoma cells. In the study by Cheung et al. [70], murine B16 melanoma cells were incubated with geoditin A for 48 h. The compound was not only cytotoxic (IC_50_ ≈ 10 μg/mL), but it also reduced tyrosinase (TYR) glycosylation and melanogenesis at sub-lethal doses (≤5 μg/mL). The effect was dose dependent but ROS (reactive oxygen species) and MITF (microphthalmia-associated transcription factor) independent.

**Table 2:** Cytotoxic activity of miscellaneous triterpenoid compounds.

Compound	Cell Line	Results		References
22*β*-hydroxytingenone	SK-MEL-28^H^	IC_50_ 4.32 μM (24 h)		[71]
		IC_50_ 3.72 μM (48 h)		
		IC_50_ 3.29 μM (72 h)		
		IC_50_ 3.2 μM (72 h)		[72]
Celastrol (CEL)	B16-F10^A^	IC_50_ 3.56 μM (48 h)		[69]
Cucurbitacin (Cuc)	A-375.S2^H^	IC_50_ 15.57 μM (24 h)		[73]
	B-16^A^	IC_50_ 65.31 μM (48 h)		
Cucurbitacin B (Cuc B)	SK-MEL-28^H^	IC_50_ 0.36 μM (24 h)		[26]
		IC_50_ 0.52 μM (24 h)		
	A375^H^	IC_50_ 1.54 μM (24 h)		
		IC_50_ 0.015 μg/mL		[33]
		IC_50_ 1.59 μM (24 h)		[22]
	B16-F10^A^	IC_50_ 0.32 μM (24/48/72 h)		[74]
Cucurbitacin D (Cuc D)	SK-MEL-28^H^	IC_50_ 0.40 μM (24 h)		[26]
	A375^H^	IC_50_ 0.32 μM (24 h)		
Cucurbitacin E (Cuc E)	SK-MEL-28^H^	IC_50_ 0.70 μM (24 h)		[26]
	A375^H^	IC_50_ 0.54 μM (24 h)		
Benzyl (2*α*,3*β*) 2,3-diacetoxy-olean-12-en-28-amide (EM2)	518A2^H^	IC_50_ 1.5 μM (72 h)		[31]
	NiH 3T3^A^	IC_50_ 33.8 μM (72 h)		
Erigeronol	B16^A^	IC_50_ 7.77 μg/mL		[75]
Geoditin A	B16^A^	IC_50_ ≈ 10.0 μg/mL (48 h)		[70]
Ilexgenin A	B16-F10^A^	IC_50_ 27.34 μM (24 h)		[36]
		IC_50_ 12.44 μM (48 h)		
Maslinic acid (MA)	SK-MEL-2^H^	IC_50_ 14.7 µM		[9]
	518A2^H^	IC_50_ 13.7 μM (72 h)		[31]
	NiH 3T3^A^	IC_50_ 38.8 μM (72 h)		
	B16-F10^A^	FBS	NO FBS	[76]
		IC_50_ 86.22 μM (24 h)	IC_50_ 3.46 μM (24 h)	
		IC_50_ 42.0 μM (24 h)		[77]
		IC_50_ 38.07 μg/mL (72 h)		[78]
Tyramine-MA conjugate	B16-F10^A^	IC_50_ 8.06 μg/mL (72 h)		[78]
Xanthoceraside	A-375.S2^H^	IC_50_ 5.71 μM (24 h)		[79]
Taraxasterol	SK-MEL-28^H^	Cell viability: ~70% (C = 20 μg/mL) (24 h)		[68]
		Cell viability: ~60% (C = 20 μg/mL) (48 h)		
		Cell viability: ~40% (C = 20 μg/mL) (72 h)		
	A375^H^	Cell viability: ~75% (C = 20 μg/mL) (24 h)		
		Cell viability: ~55% (C = 20 μg/mL) (48 h)		
		Cell viability: ~45% (C = 20 μg/mL) (72 h)		

The letters in superscript next to the cell line name mean A—animal cell line, H—human cell line. Cell lines: SK-MEL-28 human malignant melanoma; B16F10, B16 murine melanoma; A375.S2, A375 human malignant melanoma; 518A2 human melanoma; NiH 3T3 murine Swiss NIH embryo; SK-MEL-2 human malignant melanoma. Abbreviations: IC_50_—median inhibitory concentration, FBS—foetal bovine serum.

## 4. Mechanisms of Cytotoxic Activity of Natural Triterpenoids

Antimelanoma potential of triterpenoids involves a number of different mechanisms, which finally lead to cell death. This may be due to either apoptosis stimulation or non-apoptotic cell death pathways. The most important mechanisms reported for triterpenoids, with respect to their antimelanoma effect, are summarized in Table 3.

Most of the reviewed triterpenoids exerted activity via an apoptosis extrinsic, mitochondrial-mediated pathway, while none of the described compounds stimulated an intrinsic pathway of apoptosis. Triterpenoids also caused cell cycle arrest, by cyclin D1, D2 down-regulation, p21 up-regulation or CDK2 (cyclin-dependent kinase 2) inhibition.

Autophagy is a process of catabolizing cellular components by the cell itself and is claimed to be one of the possible ways to inhibit tumour growth. A single study showed an interesting mode of action of ganoderic acid in three human melanoma cell lines (HT-144, 1359-mel and DM-331), which involved both apoptosis and autophagy. The compound not only induced an apoptotic process in the cells, but also initiated a transfer of signals, characteristic of both processes, such as an increase in Beclin-1 and LC3 proteins, and their interaction with apoptotic and/or anti-apoptotic molecules in melanoma cells [83].

Moreover, triterpenoids also affected the cytoskeleton integrity, which is crucial for cell division and motility, and this was especially noticeable for LU [57,103], which caused actin stress fibre disassembly in murine B16F10 melanoma cells. Some of the triterpenoids were also involved in the inhibition of melanoma cell migration ability, by decreasing the production of metalloproteinases, *β*-catenin or inhibition of haptotaxis [98]. Alqathama et al. [64] described an interesting synergy between UA and a flavonoid—quercetin. Both compounds significantly decreased murine B16-F10 melanoma cell migration in the wound-scratch assay, at lower doses than when administered alone [83]. Moreover, LU, UA and CEL inhibited angiogenesis by the inhibition of capillary formation and endothelial cell proliferation [32,95,97] in murine B16-F10 melanoma cells.

One of the most widely studied cucurbitacins, Cuc B, inhibited the growth of several human malignant cells, including melanoma cells. In studies on the mechanism of action, Cuc B was found to inhibit ERK ½ phosphorylation [104], while another study showed that ERK ½ is activated by this compound [105]. Most probably, cucurbitacins not only inhibits phosphorylated and total ERK, but the whole MAPK kinase pathway [26].

Some of the reviewed triterpenoids also had additional impact on melanoma cells. The inhibition of melanin synthesis was observed for geoditin A, LU, (23*R*,24*E*)-acetoxymangiferonic acid and BT [57,70,93,98,100], manifested by the down-regulation of melanogenic proteins, suppression of melanin accumulation, inhibition of *α*-melanocyte-stimulating hormone and other mechanisms (see Table 3 for details). Anti-inflammatory [101,102] and antioxidant [76,77,95] activity of triterpenoids was also reported, and this can be yet another advantage of these compounds in combating melanoma, as cancer processes are often related to inflammation state or oxidative damage.

Studies conducted by Kaneda et al. [100] focused on the antimelanoma activity of an interesting tetracyclic triterpenoid (23*R*,24*E*)-23-acetoxymangiferonic acid (23*R*-AMA) and its mechanism of action. This cycloartane triterpenoid occurs in *Garcinia* sp. bark. The results indicated that this compound reduced melanin production by inhibiting tyrosinase expression, which is a key enzyme in melanin synthesis. The compound also inhibited MITF (melanocyte inducing transcription factor) in the B16-F10 melanoma cell line, the factor that not only regulates melanin production, but is also responsible for cell cycle control, proliferation, survival and migration. Moreover, 23*R*-AMA was also found to completely inhibit the activity of *α*-MSH ([Nle4, d-Phe7]-*α*-melanocyte-stimulating hormone tri-fluoroacetate salt)/IBMX (3-isobutyl-1-methylxanthine), which at the concentrations over 12.5 μg/mL induced intracellular melanin accumulation in B16-F10 melanoma. The compound was more potent than arbutin, used as a positive control. No significant change in apoptosis-related factors such as Bcl-2 family protein, and autophagy indices (mTOR and LC3-II) was observed, which indicates that 23*R*-AMA-induced growth inhibition differs from apoptotic mechanisms generally observed in cell death. These results suggest that the total protein reduction by 23*R*-AMA is not due to the induction of apoptotic cell death and autophagy but is rather associated with inhibition of proliferation.

## 5. Strategies to Improve the Bioavailability of Selected Natural Triterpenoids as Antimelanoma Agents

Triterpenoids, despite their high therapeutic potential, have poor water solubility, which is a highly unfavourable feature in terms of their potential use as therapeutic agents. This problem is clearly reflected in the limited number of clinical trials reported. One of the strategies applied to solve these difficulties is the use of new drug delivery systems, that can significantly improve the solubility, stability and also bioavailability of these compounds, thereby promoting their antitumour effect and facilitating their use in therapy. Importantly, in 2020, a review article was published which in-depth described various delivery systems for birch bark-derived triterpenoids; the article describes methods such as the use of nanoparticles, polymer matrices, or encapsulation/emulsification in lipophilic media [106]. In this section, some of the studies that were aimed to improve the solubility and bioavailability of selected natural triterpenoids with a view to improve their antimelanoma potential, are summarized.

### 5.1. Cyclodextrin Complexation

#### 5.1.1. In Vitro Experiments

Cyclodextrins (CDs) are oligosaccharides with a hydrophilic outer surface and a hydrophobic interior, which can accommodate a large variety of drug molecules [107]. Due to their low immunogenicity and toxicity, CDs are promising candidates for biomedical applications [108]. Pentacyclic triterpenoids can form inclusion complexes with various types of CDs. This combination creates a hydrophilic matrix that facilitates the dispersion of drug molecules and thereby increases their water solubility. Therefore, this approach is very attractive, practical and efficient to improve pharmaceutical and physicochemical properties of drugs, especially those with weak water solubility and rapid metabolism, such as free triterpenoids like UA and OA [63].

The study by Soica et al. [61] described the synergistic in vitro activity of OA and UA in a complex with hydroxypropyl-*γ*-cyclodextrin (HPGCD) on A375 and A2058 human melanoma cell lines. UA and OA potentiated each other’s antiproliferative activity in vitro and also showed synergism of action. UA at concentrations of 85 and 100 μM showed an antiproliferative effect on the A375 cell line, while concentrations of 60 and 75 μM exhibited the strongest antiproliferative effect on A2058 cells. Incorporation of UA into HPGCD slightly increased the activity of this compound on both cell lines, but only at the concentration of 85 μM was this effect statistically significant. OA also exerted antiproliferative activity on both A2058 and A375 human melanoma cell lines, with IC_50_ values of 60 and 75 μM, respectively. Complexation with HPGCD also led to a slightly increased activity of OA. The UA:OA mixture (1:1) showed antiproliferative activity on both cell lines, with the strongest effect seen at the concentration of 100 μM. Data obtained from this study suggest a synergy between these two triterpenic acids, and complexation with cyclodextrin sustains this synergic effect and slightly improves the activity of these two compounds. Moreover, water solubility was improved as well as bioavailability.

Another research study conducted by the same authors was performed on the UA and OA inclusion complexes with CDs. The 2-hydroxypropyl-*β*-cyclodextrin (HP*β*CD) and 2-hydroxypropil-*γ*-cyclodextrin (HP*γ*CD) were selected as host molecules. The antimelanoma potential of the complexes was evaluated on three melanoma cell lines: human A375 and Sk-MEL-2 and murine B164A5 cells, and compared to the results of antiproliferative activity of UA and OA alone. The results showed an increased antiproliferative activity of the two acids complexed with cyclodextrins against all three melanoma cell lines. UA complexes with both HP*γ*CD and HP*β*CD exerted a significant antiproliferative activity, but HP*γ*CD proved to be the most promising inclusion partner for UA. On the other hand, weaker results were observed for OA complexes, where the most promising inclusion partner was HP*β*CD. The incorporation of these two active compounds inside hydrophilic CDs proved to be a good option to increase their antiproliferative activity [63].

BA solubility also can be increased by complexation with cyclodextrin. According to various studies, BA, as well as BT molecules, fit best inside *γ*-cyclodextrin cavity as well as its derivative hydroxypropyl-*γ*-CD (HPGCD). BA water solubility increased 14 times when in complexes with HPGCD and this has also improved its antimelanoma activity as shown on the B16 cell line [4]. Soica et al. [51] described BA activity in complexes with octakis-[6-deoxy-6-(2-sulfanylethanesulfone)]-*γ*-CD (GCDG) on metastatic and non-metastatic B164A5 melanoma cell lines. The results indicated that the use of GCDG alone did not have any significant effect on B164A5 cell proliferation. In contrast, GCDG cyclodextrin complexed with BA led to increased antiproliferative activity, but this increase was not statistically significant. After 72 h of incubation with this complex, 50.30% of the non-metastatic B164A5 cells, in comparison to 42.33% of the metastatic B164A5 cells, were viable.

#### 5.1.2. In Vivo Experiments

In vivo activity of OA and UA in a complex with HPGCD was tested in experiments with mice, divided into four groups: a control group, an OA:HPGCD-treated group, a UA:HPGCD-treated group and an OA:UA:HPGCD-treated group. After 0.5 h of treatment, the animals were exposed to 7,12-dimethylbenz(a)anthracene (DMBA) and UVB radiation (carcinogens). Mice treated with OA:UA:HPGCD complex showed a very small difference in the progression of erythema after 6 weeks of treatment, while the control group showed an important change (more than 230 units). A slight, non-linear increase in the melanin content without statistical significance was observed in all groups [61].

Another in vivo study on complexation with CDs was performed using C57BL/6J mice with developed melanoma. BA complexed with GCDG was administered daily at a concentration of 100 mg/kg body weight. The control group was inoculated with B164A5 cells and the study group was inoculated with B164A5 cells and treated with the above-mentioned complex. The study showed that the BA:GCDG complex visibly reduced tumour weight and volume, whereas smaller values for erythema and melanin levels, as well as a TEWL parameter, an indicator of transepidermal water loss, were observed in the treated group, as compared to the control group. All these data suggest that the BA:GCDG complex has significant influence on the melanoma treatment [51].

### 5.2. Nanoparticles and Microspheres

#### 5.2.1. In Vitro Experiments

Metallic nanoparticles can interact with biologically active molecules, which is used for diagnostic and treatment purposes. Silver nanoparticles (AgNPs) have antibacterial and cytotoxic properties, due to their ability to generate DNA damage, cell cycle arrest and cause oxidative stress, which in turn leads to apoptosis and finally to necrosis [109,110].

In a study by Danciu et al. [43], BT silver nanoparticles (AgNPs) were tested for an antimelanoma effect. Bare and PEG (Polyethylene Glycol)-capped AgNP-Betulin (AgNP-B) formulations were synthesized and examined against melanoma B164A5 and B16Ova cells, normal human keratinocytes HaCaT and primary epidermal melanocytes HEMa. Dose–response experiments were performed using different concentrations (0, 0.5, 1, 5, 15, 30, 50 and 100 μM) and 72 h exposure, with BT as a positive control. BT showed a dose-dependent effect and was highly toxic to B164A5 melanoma cells at concentrations > 15 μM, viability rate was lower than 9% (IC_50_ 4.269 μM), with a similar effect on B16Ova cells (IC_50_ value 3.89 μM). In the case of AgNP-B formulation, the IC_50_ value was 0.9301 μM, significantly lower than the one obtained for BT, what proves the potent inhibitory effect of AgNP-B on B164A5 melanoma cell viability. This formulation showed a strong dose-dependent decrease in B164A5 melanoma cell survivability percentage of around 60%, even at the concentrations as low as 1 μM. Concentrations higher than 50 μM led to a viability rate of 5%. The B16Ova cells turned out to be less sensitive to the effect of AgNP-B, with the IC_50_ 20.26 μM, higher than for BT (3.89 μM). The PEGylated formulation of AgNP-B induced a significant reduction in the viability of both B16Ova and B164A5 cells, with IC_50_ 5.74 and 2.47 μM, respectively. Bare AgNP with IC_50_ 70.81 μM caused a decrease in B164A5 cell viability at concentrations higher than 15 μM, while the highest concentration tested was 100 μM and this affected almost all cells. AgNP also reduced the percentage of viability in the case of B16Ova cells; the result was similar to that obtained for B164A5 cells (IC_50_ 73.07 μM). A similar effect of PEG-AgNP-B against B16Ova cells in comparison with BT, and stronger effect as compared to AgNP-B formulation was observed in the case of B164A5 cells, where both formulations were more potent than BT. The impact of the tested compounds on cellular viability was also examined on normal human cell lines: HEMa and HaCaT. The AgNP formulations exerted over a 50% reduction in cell viability, at 30 μM in HEMa cells and at 50 μM in HaCaT cells, suggesting a susceptibility of human melanocytes to AgNPs; nevertheless, the formulation was less toxic as compared with BT. At concentrations higher than 15 μM, AgNP-B caused a significant decrease in cell viability both in the case of HEMa and HaCaT cells, with the IC_50_ 25.72 and 39.73 μM, respectively, while the IC_50_ values for BT were 24.70 for HaCaT and 21.63 μM for HEMa. PEGylated formulations of BT showed over a 50% reduction in viable cell percentage at concentrations > 30 μM for HaCaT (IC_50_ 86.83 μM) cells and >15 μM for HEMa cells (IC_50_ 52.98), which is higher than for BT. Thus, PEG-AgNP-B formulation was less toxic in comparison to BT. The data provided by this study clearly indicate that bare and PEGylated silver nanoparticles loaded with BT meet the basic criteria for safe use, show adequate anticancer properties and are less toxic than BT. On the other hand, in the study by Ghiulai et al. [111], BA was combined with gold nanoparticles (BA-GNP) and showed selective cytotoxic activity for cancer RPMI-7951 cells. BA-GNP and BA were safe against normal HaCaT cells, and only at higher concentrations (50 μM) induced low toxicity (87.3% and 86.9% of cell viability for BA and BA-GNP, respectively). Importantly, the combination of BA with gold nanoparticles at concentration of 25 and 50 μM resulted in a stronger cytotoxic effect (75.1% and 63.4% of cell viability, respectively) compared to BA at the same concentrations used alone (89.6% and 85% of cell viability, respectively). It is worth noting that only the highest concentration of BA-GNP (50 μM) significantly inhibited the viability of RPMI-7951 cells. In another study, derivatives of BA, OA and UA bearing 1,2,3-triazole moieties functionalized with gold nanoparticles and their cytotoxic activity against A375 cells were presented. The strongest effect was presented by the OA derivative (OA-HOBt-loaded nanoparticles) at a concentration of 50 μM (59.3% of cell viability), while the weakest was the UA derivative (UA-HOBt-loaded nanoparticles) (74.8% of cell viability). No cytotoxicity was observed against HaCaT cells, and the conjugates were shown to be more active against their parent compounds, which shows that gold nanoparticles can enhance the activity of triterpenoid compounds [112].

Li et al. [69] studied celastrol (CEL)-loaded nanoparticles (CEL-NPs) with respect to their activity against melanoma B16F10 cells. CEL has been reported to have anticancer activity, unfortunately high toxicity and poor water solubility limit its use in clinical treatment. Three compounds were evaluated in the study: CEL, CEL-NPs and mPEG-PLL polymer created by CEL conjugation onto a methoxyl poly(ethylene glycol)-b-poly(L-lysine) backbone. The IC_50_ values for CEL and CEL-NPs were 3.56 and 2.81 μM, respectively; on the other hand, the mPEG-PLL polymer at all tested concentrations showed low cytotoxicity. These results indicate that spherically shaped CEL-NP particles exert excellent antitumour activity against B16F10 mouse melanoma cells, reduce side toxicities in comparison to free CEL and provide a new potential nanomedicine for antimelanoma treatment.

In one study, cucurbitacin (Cuc)-loaded poly(lactic-co-glycolic acid) (PLGA) particles of different sizes were prepared as the sustained-release system for intratumoural injection. Their physicochemical properties, particle–cell interaction, pharmacokinetics and in vitro cytotoxicity towards B16 murine melanoma cells were studied. After 48 h of incubation, raw Cuc had the lowest IC_50_ value of 65.31 μg/mL, while for large PLGA microspheres (L-MPs), small PLGA microspheres (S-MPs) and lipid nanoparticle (NP) IC_50_ values were 464.37, 283.41 and 82.94 μg/mL, respectively. The IC_50_ value of the NPs was just slightly higher than the raw Cuc and was not consistent with the regression tendency. A possible explanation for these results may be the absorption of NPs on the cell surface, which allows direct penetration of the drug into the cytoplasm. Raw Cuc was also tested on human A375.S2 cells and exhibited stronger cytotoxicity to them than to B16 cells, with an IC_50_ value of 15.57μg/mL. An interesting finding of this study was that the initial burst release of tested particles was strongly correlated with the IC_50_ values. Another conclusion that can be drawn is that the in vitro release depended on the particle size. As smaller particles induced a strong initial burst, lower IC_50_ values were obtained [73].

#### 5.2.2. In Vivo Experiments

An in vivo study using PEG-AgNPs (PEG-capped silver nanoparticles) and PEG-AgNP-B (PEG-capped betulin silver nanoparticles) was conducted in a murine melanoma model (using C57BL/6J mice inoculated subcutaneously with B164A5 melanoma cells). PEG-AgNPs were administered at doses of 10 mg/kg body weight. There were four groups of mice: (1) control, (2) mice inoculated with melanoma cells, (3) mice inoculated with melanoma cells and treated with PEG-AgNPs, (4) mice inoculated with melanoma cells and treated with PEG-AgNP-B. The results showed significant differences in the content of melanin between the control group and the remaining experimental groups. The highest values were observed in the group of mice inoculated with melanoma cells but without treatment. On the other hand, the group treated with PEG-AgNP-B showed the lowest values of erythema, while in other groups, the values were as high as in the control group. These data indicate that PEG-AgNP formulation showed a significant antimelanoma efficacy in vivo [43].

CEL-loaded nanoparticles and their in vivo antitumour effect were tested on the B16F10 murine melanoma model. Tumour growth was inhibited in the CEL-treated groups compared to the PBS (phosphate-buffered saline)-treated control group. Moreover, higher concentrations of CEL or CEL-NPs resulted in smaller tumour sizes as compared to mice treated with lower concentrations. This indicates that the effectiveness of the treatment was dose related. It is also worth mentioning that CEL-NPs showed little systemic toxicity because the body weights in the CEL-NP-treated groups were slightly lower than in the PBS and mPEG-PLL-treated groups. In the CEL-NP-treated groups the body weights were a little lower than in other groups, indicating that the CEL-NPs had some systemic toxicities. In addition, in the groups treated with free CEL, weight reduction was greater than in the CEL-NP groups, which indicates that free CEL has high toxicity [69].

In vivo experiments by Guo et al. [73] showed that both the NPs (lipid nanoparticles) and the raw Cuc did not exert a high antitumour effect, what may be related to the high toxicity of Cuc. On the other hand, the pharmacokinetic study indicated that the high drug level retained during the whole experimental period and the S-MPs (small PLGA microspheres) showed better evaluation indices than others. A pharmacodynamic study with a xenograft and homograft model showed that MPs displayed better treatment efficiency; this was attributed to their good release properties and suitable particle size. The results indicate that the intratumoural administration of Cuc-loaded PLGA showed limited and unsatisfactory antimelanoma properties in xenograft animal models. According to the authors, this could be due to the leakage of the carriers from the injection site through the pinhole, as well as an unwanted initial burst release. Further research carried out by the same authors [113] focused on the evaluation of in situ-forming implants (ISFIs) containing Cuc. Good stability, easy production and most importantly, better anticancer activity in vivo are the advantages of the ISFIs in comparison to Cuc- loaded PLGA particles. PLGA and SAIB (bioabsorbable sucrose acetate isobutyrate) were used as the matrix materials. PLGA ISFIs and SAIB ISFIs are two in situ-forming implants which were evaluated for their activity on solid tumour treatment via intratumoural injection. Tumour-bearing C57BL/6 mice were used to investigate the effect of intratumoural Cuc-loaded ISFIs on overall animal survival. In this experiment, SAIB ISFIs showed a better antitumour effect than S-MPs, although both of them had similar release profiles in vitro. Better results obtained for SAIB ISFIs may be due to their adhesive properties and their ability to reduce the leakage through the pinhole. The obtained results showed that SAIB ISFIs tested on an animal model were characterized by an appropriate release profile, and acceptable pharmacokinetic and pharmacodynamic profiles. It is also easy to manufacture and display a desirable therapeutic index due to the lower plasma drug concentration and higher drug retention in a tumour. A great advantage of an injectable depot ISFI formulation is the possibility to avoid constant drug injection or infusion; it can also minimize unwanted side effects caused by changes in plasma drug levels.

### 5.3. Ethosomes

Ethosomes have the structure of a multilayer artificially designed lipid nanovesicle; they are similar to liposomes, with the difference being that they are characterized by a high ethanol content (20*–*45%). Ethosomes are capable of encapsulating both hydrophobic and hydrophilic compounds. They can penetrate deep layers of the skin more efficiently than traditional liposomes, which means that they have greater potential in the treatment of skin diseases. It is worth mentioning that ethosomes have recently been shown to enhance the antimelanoma properties of some compounds. Ethosomes containing BA and BT were prepared. BT was the model drug for all formulations and drug-free ethosomes were also prepared. The obtained formulations were evaluated for their cytotoxicity towards B16-F10 melanoma cells. Cells were treated with the increasing concentrations of triterpenoids provided both in free form in ethanol solutions (0.1, 1, 10, 20 μM) and encapsulated in ethosomes for 48 h. BT-ethosome formulation showed an IC_50_ value of 2.43 μM, compared to 14.38 μM for BT ethanolic solution. BA formulation with ethosomes showed an IC_50_ value of 3.07 μM compared to 16.41 μM for an ethanolic solution of BA. Surprisingly, the effect of triterpenoid extract was not improved by using the ethosomal formulation, with IC_50_ 36 and 43.51 μg/mL, respectively. Nevertheless, the results of this study clearly showed the enhancement of a cytotoxic effect of isolated triterpenoids incorporated into ethosomes. Thus, it seems that ethosomes can be a promising carrier for BT and BA [1].

Thanks to the use of nanoparticles, ethosomes and complexation with cyclodextrins, triterpenoids that previously showed poor water solubility proved to be more soluble and thus showed greater bioavailability, which resulted in increased antimelanoma effects (see Table 4). Triterpenoids with modified bioavailability, such as PEGylated compounds, or those administered in nanoparticles, were generally more potent (Table 4). The B1645 mice cell line was more susceptible to BT silver nanoparticles, with IC_50_ 0.9301 μM, as well as to PEGylated formulations of BT silver nanoparticles (IC_50_ 2.47 μM). Also, 28-O-propynoylbetulin and similar compounds with modifications at C-28 more strongly inhibited the cell growth of human cell lines: C32, A2058 and G361, whereas BT itself showed an IC_50_ result of 12.4 μM against G361 human cells. The lowest values, below 2 μM, were observed for the MEL-2 human cell line. BA with cyclodextrin GCDG complex reduced B164A5 metastatic cell viability to 42.33%, while pure BA reduced to 57.89%. The same compounds tested on non-metastatic B164A5 cells decreased cell viability to 50.30% and 61.82%, for BA with cyclodextrin GCDG complex and pure BA, respectively. A formulation with BA-loaded ethosomes was also more potent against B16 F10 melanoma cells than BA alone (IC_50_ values of 3.07 and 16.41 μM, respectively).

**Figure 1:**
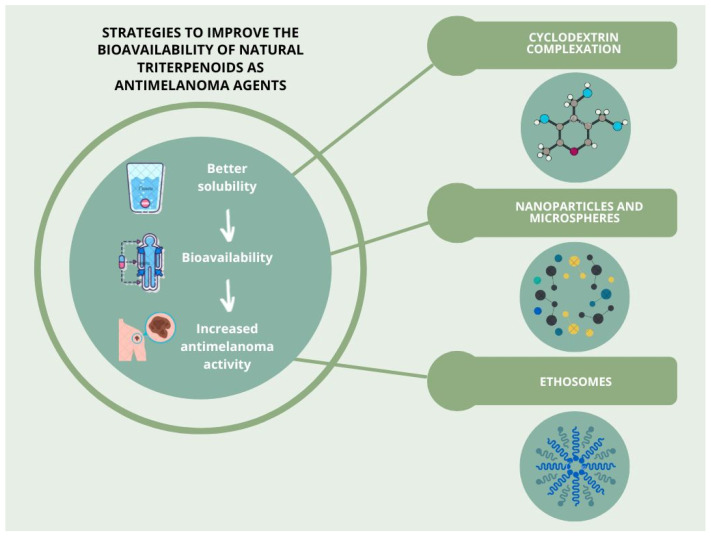
Graphical summary of the strategies to improve bioavailability and antimelanoma effect of triterpenoids.

Drug delivery systems like cucurbitacin-loaded poly(lactic/glycolic) acid particles for intratumoural injection and in situ-forming implants containing Cuc are also worth mentioning, because they provide promising opportunities to fight tumours, but further studies are certainly needed to prevent the leakage of the carriers from the injection site or unwanted initial burst release. Pharmaceutical technologies can significantly improve the solubility, stability and bioavailability of pentacyclic triterpenoids, opening up an opportunity to use some natural triterpenoid compounds in the clinical environment. A graphical summary of the strategies to improve triterpenoids bioavailability is presented in Figure 1.

## 6. Chemical Modifications of Selected Natural Triterpenoids to Improve their Effectiveness as Antimelanoma Agents

Several studies on the cytotoxic activity of triterpenoids are focused on observations of structure–activity relationships. These are important for the design of novel semi-synthetic derivatives which are based on known triterpenoid scaffolds. The main goal of chemical modifications is the improvement of solubility and enhancement of activity compared to parent compounds. The cytotoxic activity of the natural triterpenoid derivatives are gathered in Table 5, while the most promising examples of chemical modifications to improve the antimelanoma effectiveness are discussed below.

### 6.1. Betulin and Betulinic Acid

BT (Compound **1**, Table 6) and BA (Compound **5**, Table 6), are classic examples of triterpenoids that show many biological and pharmacological properties, including chemopreventive and cytotoxic, but at the same time have very low bioavailability due to their poor solubility in aqueous solutions [3]. Thus, new derivatives of these compounds are widely synthesized, to obtain the structure that would retain high cytotoxic efficacy but have greater bioavailability.

**Figure 2:**
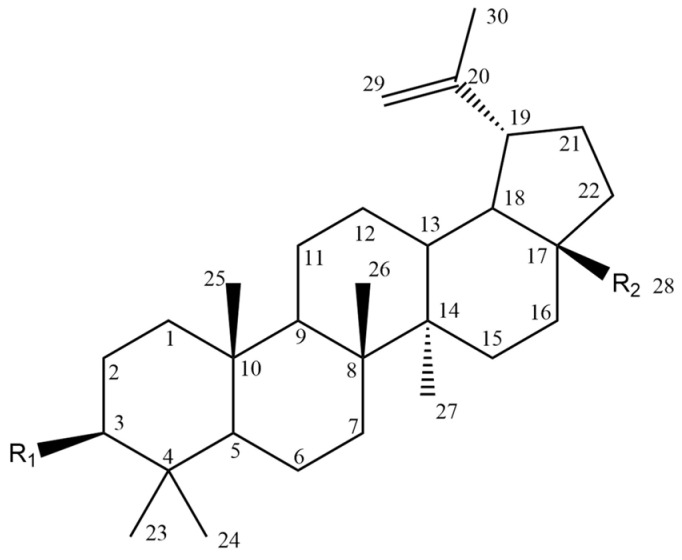
Backbone structures of betulin, betulinic acid, lupeol and their derivatives.

The BT molecule consists of one five-membered ring and four six-membered rings (Figure 2), and has three reactive moieties that include a primary hydroxyl group at C-28, an isopropenyl group at C-19 and a secondary hydroxyl group at the C-3 position [80].

To study the structure–activity relationship, carboxylic derivatives of BT, which contained an aldehyde or hydroxymethyl group at position 17, were prepared. The results showed that the carbonyl group (aldehyde or carboxylic acid) is necessary for the cytotoxicity of these compounds because while aldehydes were active, hydroxyl derivatives usually were not [46]. Other studies conducted on aldehyde derivatives of BT (betulinic aldehyde) gave comparable values of inhibition of B16 2F2 melanoma cell growth and suggested that the carbonyl function is important for the bioactivity of this compound [47]. In the study by Bębenek et al. [114], scientists combined the structure of BT with an indole molecule, and the obtained 3-indolyl betulin derivatives were then tested for cytotoxic activity on various cell lines. The obtained results show that the betulin derivative named “EB367” (28-Hydroxy-(lup-20(29)-ene)-3-yl-2-(1*H*-indol-3-yl) acetate contained at the C-28 position a free hydroxyl group (Compound **25**, Table 6) reduced the viability of both C32 and A375 melanoma cell lines by about 30–35%.

Many studies focused on the activity of BT derivatives resulting from modification of the substituent at position 28. One of the tested compounds was 28-O-propynoylbetulin (Compound **2**, Table 6). In the studyby Orchel et al. [89], the ability of both BT and 28-O-propynoylbetulin to induce apoptosis and cell growth arrest was studied. The treatment of G-361 melanoma cells with BT caused significant caspase-3 activation. Interestingly, incubation of the cells with 28-*O*-propynoylbetulin led to a nearly two times higher increase in apoptosis. It seems that this increase in potency may be related to specific structural features. In 28-*O*-propynoylbetulin, the carbonyl group is very close to the terminal triple bond which forms a relatively reactive configuration. In another study, melanotic A2058 and amelanotic C32 cell lines were incubated with various concentrations of 28-*O*-propynoylbetulin (0.1, 0.3, 1, 3, 10 μg/mL). The compound significantly inhibited the proliferation of the tested cell lines, especially at higher concentrations (3.0 and 10.0 μg/mL). 28-*O*-propynoylbetulin at the concentration of 3 μg/mL reduced the growth of A2058 cells by 45.9% as compared to the control. At the same concentration, the number of C32 cells was also reduced and amounted to 28.8% of the control. At the concentration of 10.0 μg/mL, the tested compound decreased the C32 cell number to 3.6% of the control, while more resistant A2058 cells were reduced to 10.3% of the control [84].

**Figure 3:**
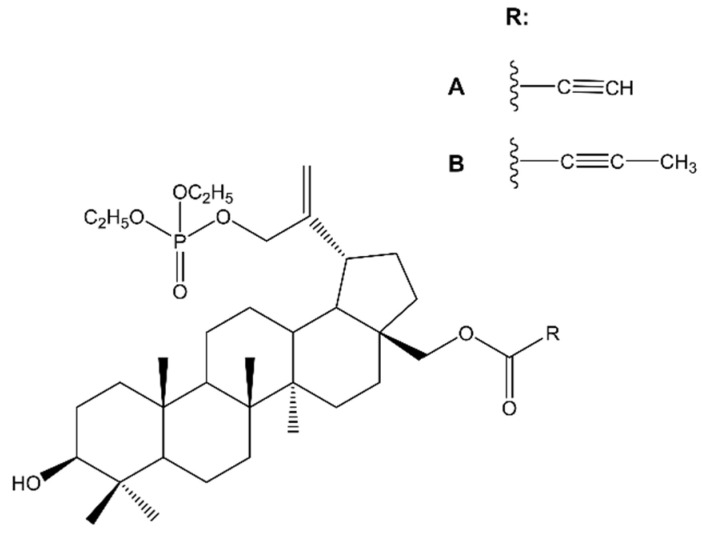
Chemical structure of 30-phosphate derivatives of betulin: 30-Diethoxyphosphoryloxy-28-propynoylbetulin (A) and 28-(2-butynoyl)-30-diethoxyphosphoryloxybetulin (B).

Chrobak et al. [44], in turn, synthesized various 30-phosphate derivatives of BT, whose antimelanoma activity was also confirmed against C-32 cells. Among various different derivatives, two compounds showed the strongest cytotoxic activity, 30-diethoxyphosphoryloxy-28-propynoylbetulin and 28-(2-butynoyl)-30-diethoxyphosphoryloxybetulin (Figure 3). The authors confirmed that these compounds have good binding interactions in comparison with the standard cytotoxic drug erlotinib. It is worth noting that the cytotoxic effect of BA alone (IC_50_ 15.61 μM) was lower than the synthesized compounds (IC_50_ 0.8–2.15 μM), more importantly, their activity was also better compared to the reference drug, which was cisplatin (IC_50_ 4.29 μM). The results of this study indicate that phosphate substituents at C-30 of BT are promising candidates for new active antimelanoma compounds.

Bębenek et al. [80] tested an acetylenic derivative, 28-*O*-propargyloxycarbonylbetulin (Compound **3**, Table 6), which at concentrations of 10.0 and 20.0 μg/mL significantly inhibited G-361 cell proliferation (compared to control). A similar effect was observed when cells were incubated with another derivative, 28-*O*-(3-butynyloxycarbonyl)betulin (Compound **4**, Table 6). Also, monoesters like 28-*O*-propynoylbetulin, 28-*O*-propargyloxycarbonylbetulin and 28-*O*-(3-butynyloxycarbonyl)betulin exhibited a more potent proapoptotic and cytotoxic effect against G-361 melanoma cells. Thus, it can be concluded that substitution at position C-28 has a significant effect on the activity of BT derivatives. These studies also have shown that a hydroxyl group at position C-3 is fundamental for the cytotoxic activity of acetylenic derivatives of BT.

In the study by Drąg-Zalesińska et al. [3], cytotoxic properties of BT and BA against the malignant melanoma Me-45 cell line were compared with five amino acid esters of BT. All the tested derivatives were much more soluble in water than their precursors, and were synthesized using natural lysine (Compound **6**, Table 6) and three lysine derivatives: (2*S*)-2,4-diaminobutanoic acid (L-Dab-OH) (Compound **7**, Table 6), (2*S*)-2,3-diaminopropanoic acid (L-Dap-OH) (Compound **8**, Table 6), L-ornithine (L-Orn-OH) (Compound **9**, Table 6) and alanine (L-Ala-OH) (Compound **10**, Table 6) which was a negative control (amino acid without an amine group in the side chain). A significant increase in apoptotic nuclei and higher cytotoxicity after 72 h incubation was seen for BT derivatives containing lysine and ornithine, as well as for diaminopropanoic acid when compared to BT and BA alone. It is worth mentioning that the length of the side chain of the L-amino acid was positively associated with improved proapoptotic and cytotoxic effects of BT esters against the Me-45 cells.

BA, which is closely related to BT, has been extensively investigated for its cytotoxic activity. As mentioned earlier, its main disadvantage is poor water solubility, which severely limits its application in vivo. For this reason, significant effort has been put into the synthesis of BA derivatives that would have similar or even higher activity and better water solubility [87].

Different studies showed that the isopropenyl chain and carboxyl group appear to be structural elements crucial for the high cytotoxic activity of BA. The carboxyl group is important for membrane permeability and consequently for the bioactivity of this compound [47]. BA derivatives can be obtained by entering modifications at numerous positions, for example, C-3, C-20 or C-28. Modifications at C-3 and C-28 were found to be promising while C-20 derivatives did not enhance cytotoxicity in tests on several cancer cell lines [87]. A slight increase in cytotoxic potency was seen when the C-3 position of BA was modified using acetic anhydride (Compound **11**, Table 6) [48]. The BA derivatives 3-oxo-23-hydroxybetulinic acid and 23-hydroxybetulinic acid also gave promising results in experiments on murine melanoma cells [49]. Further modification at the C-3 position, which gave dimethylsuccinyl betulinic acid (Compound **12**, Table 6), had an impact on the mechanism of action. BA itself is a proteasome activator, while its derivative acts as a proteasome inhibitor [87]. Conversion of the C-3-hydroxyl to a chloro-substituted acetyl group gave an extremely cytotoxic derivative (Compound **13**, Table 6) that proved to be two to four times more potent than BA itself. The IC_50_ value of 3-*O*-chloroacetylbetulinic acid against the 518A2 human melanoma cell line was as low as 8.44 ± 0.38 μM [48].

In an interesting study by Saha et al. [50], BA was combined with a DCA (dichloroacetic acid) to form a derivative esterified at the C-3 hydroxyl*—*Bet-CA (Compound **14**, Table 6), with improved activity and increased water solubility. Moreover, the compound was proven to selectively affect cancer, but not normal cells. In an in vivo study, B16-F10 cells were subcutaneously injected to female BALB/c mice to form a primary tumour. Next, Bet-CA was administered intravenously at a dose of 2.5 mg/kg body weight. On the 25th day, experimental and control mice were sacrificed and then the tumour volume was measured. The results showed that Bet-CA significantly inhibited tumour growth in comparison to DCA or BA alone, as well as their mixture: DCA + BA (1:1). The obtained ester derivative of BA, Bet-CA, might be considered a capable drug candidate as it inhibits tumour growth in vivo and cancer cell proliferation in vitro without any toxic manifestations.

Similarly to BT, BA amino acid conjugates at C-28 also showed better water solubility and had enhanced cytotoxicity [123]. On the other hand, introducing methyl ester at the same position (Compound **15**, Table 6) led to depleted cytotoxicity [48]. The above data suggest that by changing the substituents at the C-3 and C-28 positions, the activity of BA can be significantly affected.

Chatterjee et al. [45] investigated the influence of modifications introduced during the microbial transformation (with the use of Bacillus megaterium) of BA at positions C-1, C-3, C-7, C-11 and C-15 on activity against Mel-1 and Mel-2 human melanoma cell lines. Hydroxylation at C-7 and C-15 in BA metabolite caused a decrease in cytotoxic activity against both Mel-1 and Mel-2 cells. Oxidation at C-1, C-3 and C-11 resulted in a significant increase in the activity against Mel-2 cells, while the oxidation of the same groups of BA caused a significant decrease in activity in relation to Mel-1 cells.

**Figure 4:**
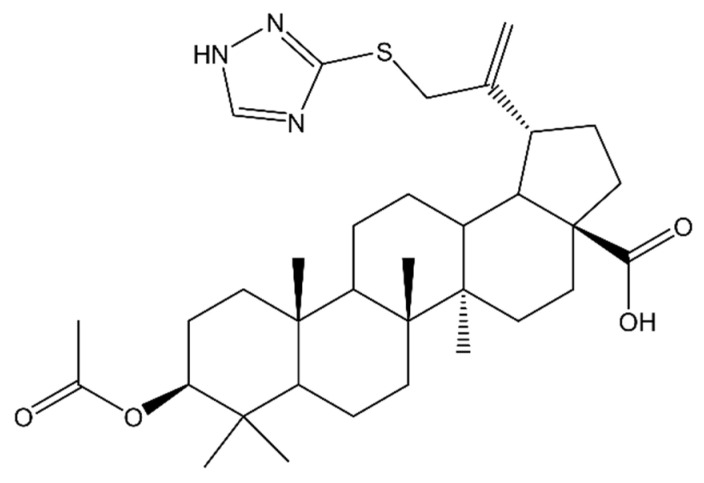
Chemical structure of acetyl-30-(1*H*-1,2,4-triazole-3-ylsulfanyl)-betulinic acid (BA-TZ).

In another study, betulinic acid derivative was synthesized in which a 1,2,4-triazole substituent was introduced at the C-30 carbon, and an acetyl group was introduced at the C-3 position: acetyl-30-(1H-1,2,4-triazole-3-ylsulfanyl)-betulinic acid (BA-TZ) (Figure 4). The compound showed a significant cytotoxic effect by reducing the viability of RPMI-7951 cells up to 24.5% (50 µM). In comparison, 5-FU at a concentration of 10 µM reduced cell viability to 59.66%. Relative to normal HaCaT cells, the compound showed no toxicity at concentrations up to 10 µM. Therefore, it can be concluded that BA-TZ is a promising candidate for an antimelanoma drug with strong cytotoxic activity and selectivity of action [115].

**Figure 5:**
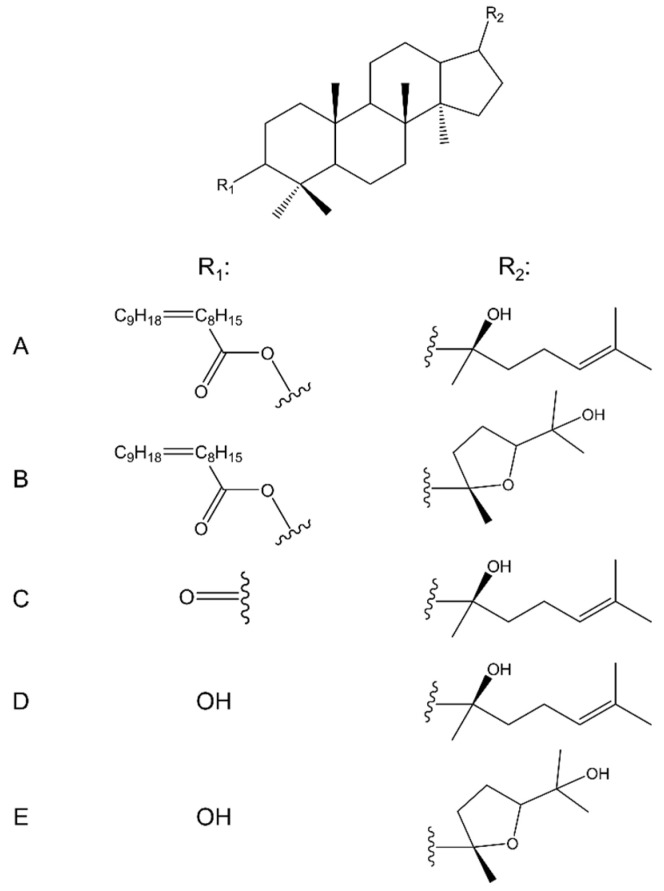
Chemical structure of 3β-oleate-20*S*-hydroxydammar-24-en (**A**), 3β-oleate-20*S*,24*S*-epoxy-25-hydroxydammarane (**B**), 20*S*-hydroxydammar-24-en-3-on (**C**), 3β,20*S*-dihydroxy-dammar-24-en (**D**) and 20*S*,24*S*-epoxy-3β,25-dihydroxydammarane (**E**).

In a study by Farabi et al. [121], researchers isolated from the stem bark of Aglaia elliptica (C.DC.) two new dammarane-type triterpenoid compounds: 3β-oleate-20*S*-hydroxydammar-24-ene (Figure 5A) and 3β-oleate-20S, 24*S*-epoxy-25-hydroxydammarane (Figure 5B), and the well-known 20*S*-hydroxydammar-24-en-3-one (Figure 5C). Moreover, as a result of the transesterification of two new compounds, two synthetic derivatives were obtained: 3β,20*S*-dihydroxy-dammar-24-ene (Figure 5D) and 20*S*,24*S*-epoxy-3β,25-dihydroxydammarane (Figure 5E). These compounds were then tested for cytotoxic activity on B16-F10 cells. The most active was 20*S*-hydroxydammar-24-en-3-one, a result of which the researchers concluded that the ketone group at C-3 plays an essential role in the cytotoxicity of this type of compound. In turn, 3-oleate-20*S*-hydroxydammar-24-ene and 3-oleate-20*S*,24*S*-epoxy-25-hydroxydammarane showed significantly weaker activity, which indicates that the presence of fatty acid in the dammarane system reduces its cytotoxic effect.

**Figure 6:**
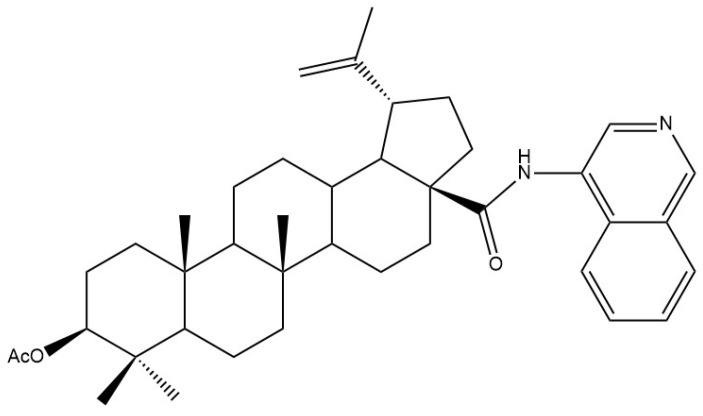
Chemical structure of 4-isoquinolinyl amide of 3-*O*-acetyl-betulinic acid.

Among the various amide derivatives of betulinic acid synthesized by Hoenke et al. [116], the 4-isoquinolinyl amide of 3-*O*-acetyl-betulinic acid (Figure 6) showed particular activity. It showed the highest cytotoxicity on A375 cells (EC_50_ ¼ 1.48 µM) with no toxicity on non-malignant NIH 3T3 fibroblasts, and the selectivity index was >91.2, which shows that it is a promising candidate for a new antimelanoma compound.

**Figure 7:**
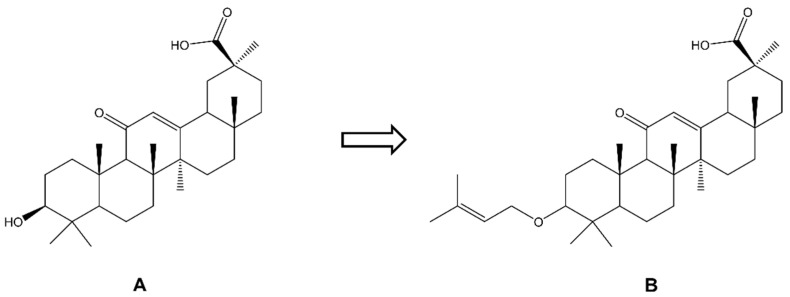
Chemical structure of glycyrrhetinic acid (**A**) and its derivative 3-*O*-prenyl glycyrrhetinic acid (NPC-402) (**B**).

In one of the studies, various derivatives of glycyrrhetinic acid found in the plant Glycyrrhiza glabra were synthesized, of which the most active against melanoma cells (A375, B16F10 and SK-MEL-28) turned out to be a derivative of 3-*O*-prenyl-glycyrrhetinic acid (NPC-402) (Figure 7). This compound was most active against B16F10 cells (IC_50_ 16 μM). After application of NPC-402 at a dose of 15 µM, a 96% death of B16F10 cells was achieved after 48 h of incubation. Additionally, this compound was not toxic to HaCaT normal human keratinocytes at concentrations <50 µM. It is worth adding that the parent compound, i.e.*,* glycyrrhetinic acid, did not affect the growth of B16F10 cells, which shows that the 3-*O-*prenyl substituent significantly increases the cytotoxic activity of this structure [122].

### 6.2. Lupeol

Lupeol (Lup-20(29)-en-3-ol, LU) (Compound **16**, Table 6) is a lupane pentacyclic triterpenoid that can be found in plants from Oleaceae and Betulaceae families [56,124]. Apart from cytotoxic activity, LU exhibits strong antioxidant- and antitumour-promoting activity [56]. Because of non-polar skeleton, LU shows low water solubility and bioavailability. The most investigated LU derivatives were its modifications at C-3 and C-20 positions, or at C-17 and C-30 positions.

Enzymatic acylation at C-3 is common in natural sources. In plants, such LU esters often accompany LU itself. LU acetate (Compound **17**, Table 6) was found to inhibit the growth of melanoma, and substitution with a succinyl moiety (Compound **18**, Table 6) at C-3 resulted in even stronger antitumour activity. Other esters, like cinnamate (Compound **19**, Table 6), palmitate (Compound **20**, Table 6) or linoleate (Compound **21**, Table 6), do not affect antitumour or anticancer activity [91]. The research on synthetic derivatives of LU is focused mainly on modifications at the C-3 position. As the literature data showed, esterification of this triterpenoid results in enhanced efficacy as compared to the parent compound. This is most probably the effect of the increased penetration and retention ability in the cell membrane. Moreover, oral bioavailability is thus improved [125]. Some reports indicate that the ketone moiety (Compound **22**, Table 6) at C-3 of the lupane skeleton plays an essential role in the melanogenic activity. Hata et al. [103] showed that oxidation of the hydroxyl group at the C-3 position of lupane results in a significant decrease in ED_50_ values and is an important factor influencing melanin synthesis in B16 F2F cells.

Even though the carbonyl group at C-17 (Compound **23**, Table 6) was found to be crucial for apoptosis induction in human cancer cells, the presence of a carboxyl group at C-17 (Compound **24**, Table 6) seems to have a strong effect on apoptosis [103]. As far as the C-20 position in LU is concerned, it seems that the terminal double bond between C-20 and C-30 is fundamental for its anticancer activity [91].

### 6.3. Oleanolic Acid

**Figure 8:**
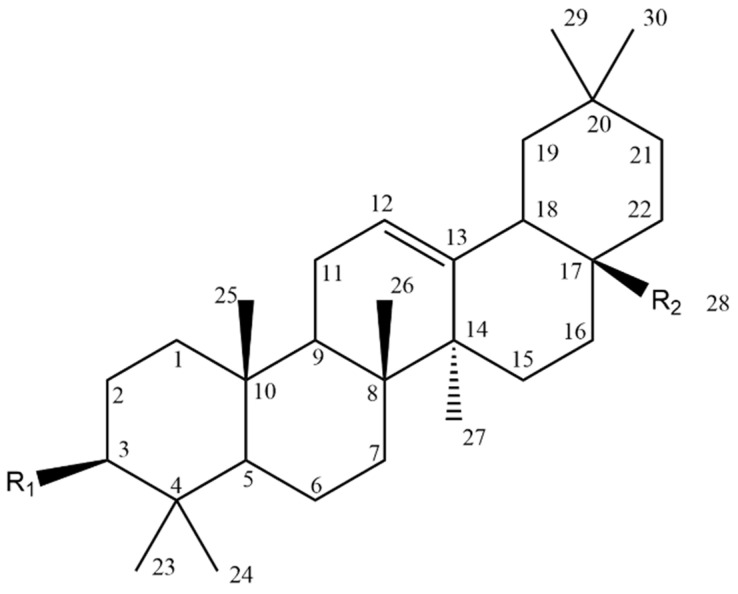
Backbone structure of oleanolic acid and its derivatives.

Oleanolic acid (3*β*-hydroxy-olean-12-ene-28-oic acid, OA) (Compound **26**, Table 7) is an oleanane-type pentacyclic triterpenoid (backbone structure is presented in Figure 8) which can be found in various plant sources, for example, from the Oleaceae family [96,124].

In recent years, many studies have focused on its antitumour effect and cytotoxicity towards a wide array of cancer cell lines, including melanoma. OA possesses three active positions, the modifications of which may affect its activity: C-3, C-28 and a double bond between C-12 and C-13. To improve the water solubility of this compound, derivatives with modifications of the hydroxyl group at C-3 and the carbonyl group at C-28 were synthesized. One of the simple synthetic derivatives was obtained by acylation at C-3. The 3-*O*-succinyl-28-benzyl oleanolic acid (Compound **27**, Table 7) was more potent than the parent compound and the benzyl-only derivative in regards to cytotoxicity and apoptotic effect. Introducing acyl groups at C-3 or C-2, or both of them, combined with the addition of the mono-/dipeptidyl group at C-28 resulted in an enhanced cytotoxic effect on B16F10 cells [126].

Du et al. [118] synthesized new MeON-Glycoside derivatives of OA by neoglycosylation (general structure: compound **29**, Table 7). Due to the large number of derivatives, we chose those with the strongest cytotoxic activity (IC_50_ < 10 µM), in which modifications were introduced at the C3 carbon: (3*S*)-*O*-(*N*-Methoxy-*N*-*α*-d-arabinosylglycyl) oleanolic acid, (3*S*)-*O*-(*N*-Methoxy-*N*-*β*-l-xylosylglycyl) oleanolic acid, (3*S*)-*O*-(*N*-Methoxy-*N*-*β*-l-lyxosylglycyl) oleanolic acid and (3*S*)-*O*-(*N*-Methoxy-*N*-d-ribosylglycyl) oleanolic acid, whose IC_50_ against A375 cells were in the range of 6–9.7 µM. In comparison, the IC_50_ of 5-FU used as the reference drug in this study was 28.5 µM.

In another study, A375 cells were treated with rhodamine B-conjugated OA derivative (RhodOA) (compound **30**, Table 7). The compound showed the ability to reduce the viability of A375 cells only after 72 **h** of incubation; however, a significant (~65–75% cell viability) effect was evident at higher doses (80–100 nM). On the other hand, in relation to normal HaCaT cells, the cytotoxic effect at the same concentrations was less pronounced (83% cell viability). A reduction in cell migration and changes such as the condensation of actin filaments and cell nuclei in A375 cells were also observed after application of the compound. These results indicate a selective antimelanoma effect of RhodOA, including a pro-apoptotic effect [119]. Heller et al. [127] also mentions that the introduction of the Michael acceptor unit into ring A of the triterpenoid skeleton increases the cytotoxic potential, e.g., OA and UA against tumour cells including 518A2 melanoma cells. Another modification at C-3 included a 3-oxo-derivative (Compound **28**, Table 7) of OA (3-oxo-olean-12-en-28-oic acid, 3-oxo-OA), which was shown to inhibit B16 BL6 cancer cell growth with IC_50_ value equal to 10.8 µg/mL [128].

**Figure 9:**
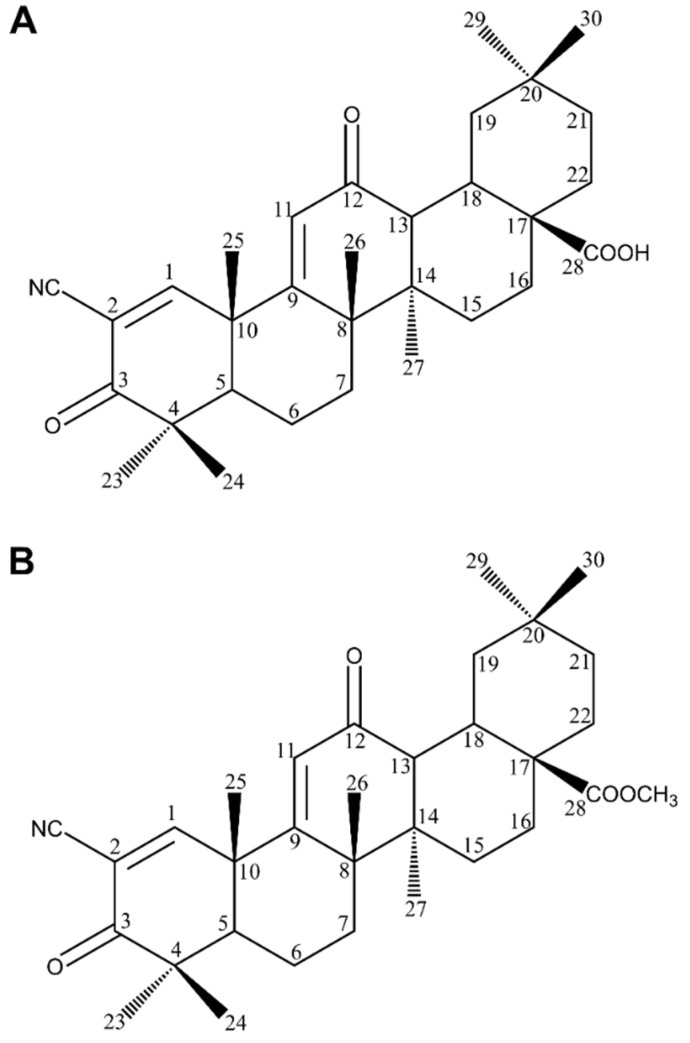
Chemical structure of (**A**) CDDO (2-cyano-3,12-dioxooleana-1,9 (11)-dien-28-oic acid) and (**B**) CDDO-Me (2-cyano-3,12-dioxo- oleana-1,9(11)-dien-28-acid methyl ester).

Promising data were obtained for two other synthetic derivatives of oleanolic acid: 2-cyan-3,12-dioxooleanane-1,9,diene-28 acid (CDDO) and CDDO-Me (Figure 9), which is CDDOs C-28 methylester. Both of them possess no functions in the rings A and C and the electron withdrawing nitrile moiety at C-2 of the A ring. These features seem to be necessary for the activity. CDDO and CDDO-Me seem to be the most potent derivatives of OA [129]. The compounds inhibited the production of NO induced by IFN-gamma factor in mouse macrophages. When investigated on B16F10, CDDO-Me showed an IC_50_ value of 5.85 mM, as compared to camptothecin—IC_50_ 2.78 mM [117]. CDDO-Me was also observed to change activation profiles of tumour-associated macrophages (TAMs), from the activation to inhibition of tumour growth, development and metastasis [11].

### 6.4. Maslinic Acid

**Figure 10:**
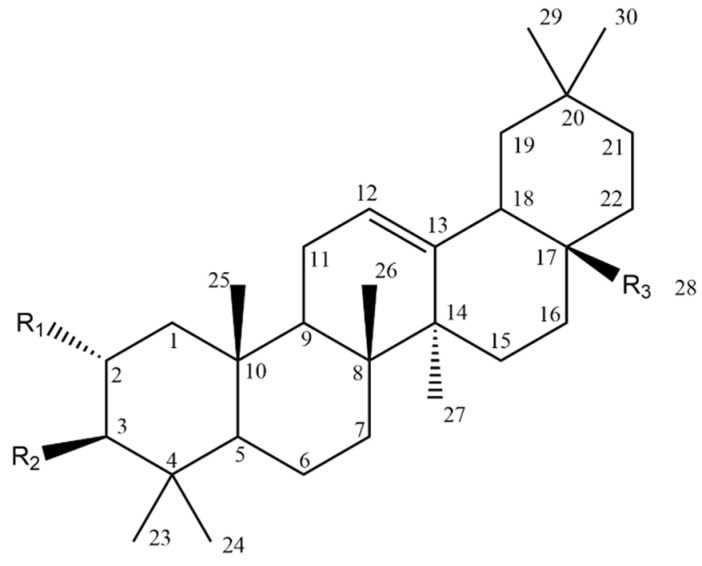
Backbone structure of maslinic acid and its derivatives.

Maslinic acid (2α,3β-dihydroxyolean-12-en-28-oic acid, MA) (Compound **31**, Table 8, Figure 10) is a characteristic triterpenoid of Crataegus oxyacantha and Olea europea, in which it is present in olive pomace oil and wax-like coatings of olive fruits [130]. This pentacyclic compound was found to exhibit many beneficial effects such as anticancer, antioxidant and anti-inflammatory effects [31,131].

To study the structure–activity relationships, different modifications of the parent compound have been investigated, including the most widely studied at C-28. Parra et al. [130] investigated almost 40 semi-synthetic derivatives of MA for their proapoptotic effect in B16F10 murine melanoma cells. All compounds with the same conjugated dicarbonylic system on ring A proved to show cytotoxicity, with at least 70% of apoptosis after 48 h. The apoptotic activity of sodium maslinate (Compound **32**, Table 8), 2,3-diacetoxy-28-cyanide MA (Compound **33**, Table 8), 28-cyanide MA (Compound **34**, Table 8) and 28-benzoyl MA (Compound **35**, Table 8) was already significant, at the lowest (1 mM) concentration after 24 h; the percentage of apoptosis was 56.67, 68.62, 78.75 and 87.50%, respectively. The authors suggest that the presence of certain substituents at C-28, especially -COONa, -CONH2, -CN and -COOBn, enhanced the apoptotic ability, as compared to the free -COOH group at C-28. It is worth noticing that there was no significant percentage of necrosis observed.

**Figure 11:**
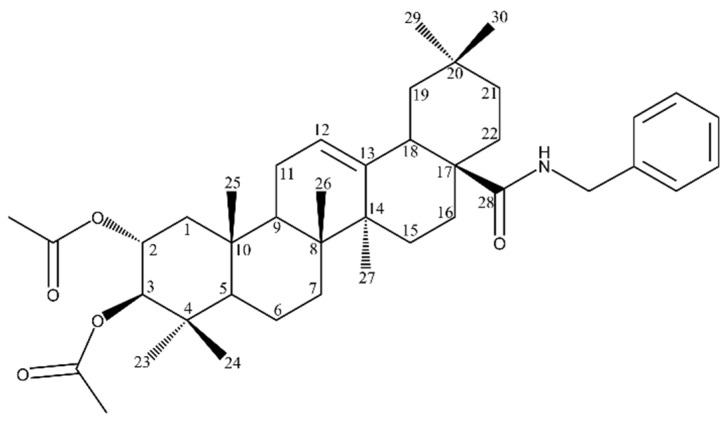
Chemical structure of EM2.

Pavel et al. [31] focused on an acetylated MA derivative: benzyl 2α,3β-diacetoxyolean-12-en-28-amide (EM2) (Figure 11). Its benzylamide structure was reported to evoke cytotoxicity in cancer cells, while being less harmful to normal fibroblasts. EM2 was reported to be more cytotoxic than the parent compound. The cytotoxicity of EM2 against 518A2 human melanoma cells reached IC_50_ 1.5 μM. EM2 also showed a concentration-dependent cytotoxicity against B164A5 and A375 melanoma cells. Importantly, EM2 was less toxic to non-malignant mouse fibroblasts, which makes it a promising chemotherapeutic and chemopreventive agent in melanoma treatment. On the other hand, Siewert et al. [132] found that introducing polar groups to C-28 positions, as in the 2-hydroxyethyl ester of MA (Compound **36**, Table 8), resulted in the lack of activity against 518A2 human melanoma cells.

### 6.5. Celastrol

**Figure 12:**
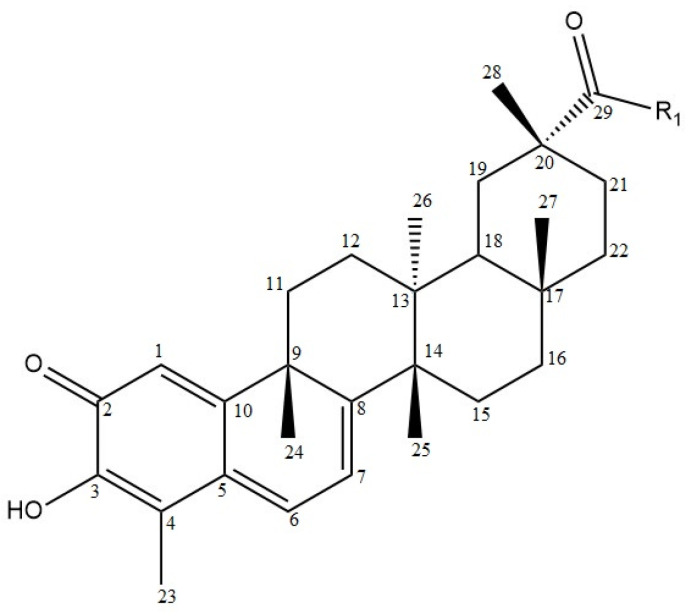
Backbone structure of celastrol and its derivatives.

Celastrol (3-hydroxy-9*β*,13*α*-dimethyl-2-oxo-24,25,26-trinoroleana-1(10),3,5,7-tetraen-29-oic acid, CEL) (Compound **37**, Table 9, Figure 12) is a quinone methide triterpenoid isolated from *Tripterygium wilfordii* with a reported antimelanoma activity [69,95]. It is also a widely studied compound, whose properties and structure–activity relationships have been described in one of the review articles [133].

Abbas et al. [134] investigated CEL and its two derivatives, pristimerin (Compound **38**, Table 9), which is a methylester of CEL, and dihydrocelastrol, which lacks quinone methide moiety, on WM115, SW1 and WM793 cell lines. Pristimerin, was equipotent or more potent than CEL in terms of inducing the apoptosis of SW1 cells. Dihydrocelastrol, on the other hand, lacked proapoptotic activity, suggesting that the quinone methide group is essential for this effect. Based on these results, the authors concluded that acidic proton in CEL does not play any role in apoptosis induction. Subsequently, ten amide derivatives modified at the carboxylic moiety were synthesized and tested on the same cell lines. Isopropyl amide (Compound **39**, Table 9), pyrrolidine amide (Compound **40**, Table 9), methoxyethyl amine (Compound **41**, Table 9), 4-methoxybenzyl amine (Compound **42**, Table 9), pyridine-3-ylmethanamine amide (Compound **43**, Table 9) and dimethyl amide (Compound **44**, Table 9) were all found to be active on SW1, while morpholine amide (Compound **45**, Table 9), monomethyl amine (Compound **46**, Table 9) and N-methylpiperazine (Compound **47**, Table 9) had no effect on any tested cell lines. As unsubstituted benzyl amide (Compound **48**, Table 9) was surprisingly found to be inactive, it was concluded that the *CO*-NH bond could be a hindering feature responsible for the reduction of cellular activity. These observations led to the synthesis of ester derivatives, such as methyl 2-hydroxyacetate ester (Compound **49**, Table 9), benzyl ester (Compound **50**, Table 9), ethyl ester (Compound **51**, Table 9) and isopropyl ester (Compound **52**, Table 9). Both benzyl and isopropyl esters showed the highest potency and ability to induce melanoma cell death. On the whole, it seems that CEL ester derivatives are more potent than amide derivatives with respect to antimelanoma effect.

### 6.6. Ursolic Acid

**Figure 13:**
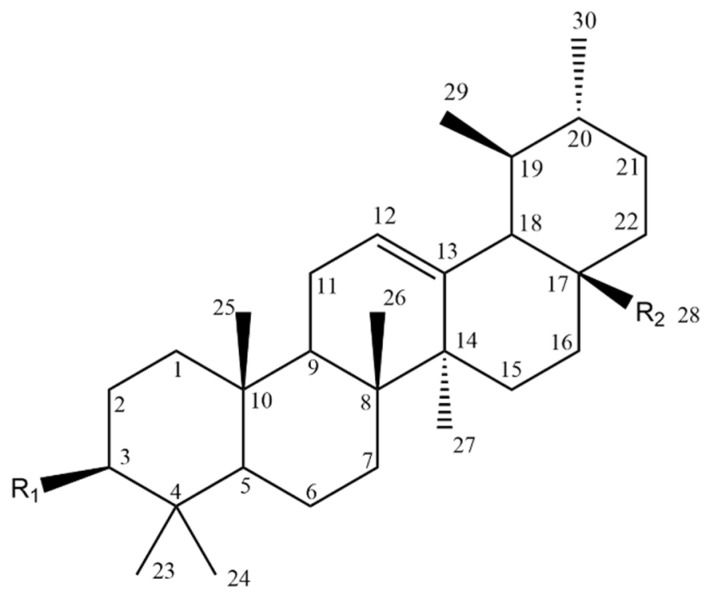
Backbone structure of ursolic acid and its derivatives.

Ursolic acid (3β-hydroxy-12-urs-12-ene-28-oic acid, UA) (Compound **53**, Table 10, Figure 13) is a pentacyclic triterpenoid acid that can be found in many plants within Lamiaceae and Rosaceae families, mainly in fruit wax coatings [63,135]. The main focus of SAR research was put on modifications at position C-3. One noticeable exception was the UA derivative (US597) with IC_50_ 8.57 μM against the murine B16F10 cell line [67].

One of simple derivatives that seems to be a promising object for further research is 3-*O*-acetylursolic acid (Compound **54**, Table 10). The compound had similar activity against the A375 cell line as UA itself. A study run by Alqathama et al. [64] showed that both UA and 3-*O*-acetylursolic acid elicit time- and concentration-dependent antiproliferative activity on A375 and HDf-a (human dermal fibroblast) cell lines (26.7 and 32.4 μM, respectively), while being selective towards normal cells (GI_50_ 89.31 and 126.5 μM for UA and its 3-*O*-derivative, respectively). This study also showed that acetyl or aminoalkyl moieties with a β*-*oriented hydrogen-bond at C-3 are more cytotoxic than the same groups attached with α-orientation.

Wang et al. [136] synthesized nine derivatives of UA, five of them were acetylated at C-3, whereas the carboxyl group at C-28 was substituted with different amide moieties (Compounds **56**–**58**, Table 10). Another four derivatives (Compounds **59**–**62**, Table 10) possessed a free hydroxyl group at C-3 and the same moieties at C-28 as C-3 acetylated compounds. The compounds with acetyl moiety at C-3 showed more potency against A375 cancer cells than derivatives with a free hydroxyl group at C-3. All compounds elicit stronger cytotoxicity than the parent compound; the most potent activity against A-375 cell line was observed for Compound **54**.

**Figure 14:**
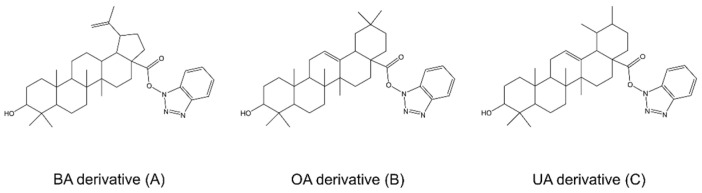
Chemical structures of benzotriazole esters of BA: 1*H*-Benzotriazole-1-yl (3β) 3-hydroxy-20(29)-lupaene-28-oate (**A**), OA: 1*H*-Benzotriazole-1-yl (3β) 3-hydroxyolean-12-en-28-oate (**B**) and UA: 1*H*-Benzotriazole-1-yl (3β) 3-hydroxyurs-12-en-28-oate (**C**).

Interesting results were presented by Mioc et al. [54] in which the benzotriazole esters of BA (Figure 14A), OA (Figure 14B) and UA (Figure 14C) were synthesized. All compounds showed dose-dependent cytotoxic activity against A375 cells, with no toxicity against HaCaT cells (results presented in Table 5). Analyses also showed pro-apoptotic activity (nucleus shrinkage, cell membrane fragmentation, decreased Bcl-2 expression and increased Bax).

## 7. In Vivo Trials

Animal trials are crucial for drug development. One of their main outcomes is the assessment of in vivo toxicity. It is noteworthy that in the majority of animal studies conducted with triterpenoids, there was no or almost no toxicity observed. On the whole, triterpenoids were shown to be promising compounds in melanoma treatment, with significant activity against tumours, metastasis inhibition and lack of toxicity.

### 7.1. Lupeol

Saleem et al. [56] inoculated 14 athymic nude mice with human melanoma 451Lu cell suspension to develop melanoma tumours. The study group was treated with 1 mg/0.2 mL LU in corn oil and the control group received solely corn oil at a dose of 40 mg per kg body weight in intraperitoneal injections three times a week. LU reduced tumorigenicity and also showed an antiproliferative effect. In another study, Bhattacharyya et al. [32] investigated LU and its combination with dacarbazine in order to assess whether they control the cancer stem cell-mediated vasculogenic mimicry and endothelial progenitor cell-mediated angiogenesis in a mouse melanoma model. The C57BL/6J mice were divided into four groups, with four mice in each group. Three groups were injected with B16-F10 cells, while the control group was injected with PBS (phosphate-buffered saline). At the time of tumour formation, animals received LU intraperitoneally, dacarbazine and their combination at doses of 40 and 80 mg/kg and both, respectively, for 7 consecutive days. Dacarbazine alone caused the instant growth of melanoma, with tumour dimensions twice as big as in the control group, which suggests resistance to this drug (dacarbazine), developing in nearly all types of aggressive forms of melanoma. The tumour treated with LU significantly decreased its dimensions, when compared to the untreated group, while the combined administration of LU and dacarbazine resulted in a less pronounced reduction of the tumour size. LU treatment also resulted in an antiangiogenic effect in the tumour, with smaller and fewer angiogenic vessels, when compared to dacarbazine. Moreover, LU treatment inhibited vasculogenic mimicry (VM), observed as the decrease in PAS-positive/CD 31-negative VM tubes. Huge CD 133+ve VM tubes were observed in the control group and dacarbazine-treated group, while the LU-treated group showed a significantly lower presence of CD 133 expressing VM.

Tarapore et al. [59] implanted 24 athymic male nude mice with human melanoma cells, with (Mel928) or without (Mel 1011) the Wnt/β-catenin signaling. Mice were divided into three groups: a control, a chemotherapeutic group and a chemopreventive group to analyze the efficacy of LU. The chemopreventive group received 40 mg/kg body weight of LU in 200 µL of corn oil in intraperitoneal injection post-inoculation, whereas the chemotherapeutic group received the same dose of LU in corn oil, administered when the tumour size reached ca. 150 mm^3^. The control group was given corn oil alone 24 h after inoculation. LU significantly inhibited the tumour growth in the LU treatment of mice with Mel 928 cells. When compared to the control, a 35% reduction in tumour size was achieved in the chemotherapeutic group and a 50% reduction in the chemopreventive group. There were no significant differences in tumour volume observed between the two treatment groups. Tumour tissue lysate examination revealed a decrease in the expression levels of Wnt target genes, localization of β-catenin and expression of proliferative markers such as PCNA (proliferating cell nuclear antigen), osteoponin and Ki-67 protein (cell proliferation marker). Immunoblot analysis showed a 13-fold reduction in the expression of VEGF in the LU-treated animals’ tumours. Moreover, the LU-treated mice showed a decrease in MMP2 (matrix metallopeptidase 2) and MMP9 (matrix metallopeptidase 9) and a correlating increase in TIMP1 (TIMP metallopeptidase inhibitor 1) and TIMP2 (TIMP metallopeptidase inhibitor 2), but no significant changes in levels of these proteins were observed in Mel 1011-implanted tumours. In toxicity exploration, LU treatment was not observed to affect body weight, food and water consumption.

In the study by Nitta et al. [137], 40 female mice C57BL bearing a B16 2F2 melanoma tumour were divided into five groups of eight mice per group: a control group, a solvent control group, animals with LU injected in the dorsal area, a control group with an injection into the tumour tissue and LU injection into the tumour tissue. Each animal was injected with 0.1 mL of LU dissolved in olive oil at a dose of 20 mg/kg body weight. Both local-injected and tumour-injected groups showed a significant decrease in tumour growth in comparison to the control group, with a bigger decrease observed in the tumour-injected group. However, no significant differences were observed in groups with dorsal and tumour tissue injection. The group with the subcutaneous administration of LU showed a reduction of Ki-67-positive areas, PCNA-positive areas, compared to the control (subcutaneous injection of olive oil) and non-treated group. Injections of LU into tumour tissues resulted in a decrease in Ki-67-positive areas, PCNA-positive areas, in comparison to the control group (injection of olive oil into tumour tissue) and non-treated group; however, no differences between both LU injections were found.

### 7.2. Ursolic Acid

UA inhibited the growth of microvessels from rat thoracic aorta in animals with induced B16 F10 melanoma. In addition, UA significantly reduced the number of capillaries directed to the tumour*—*the tumour bearing animals showed on average 12.67 ± 1.2 capillaries in comparison to the control group*—*with an average of 30.14 ± 1.35. On the 24th hour and on the 9th day after, tumour induction blood was collected to estimate levels of VEGF, NO, TIMP-1 and IL-2 (interleukin-2). In samples of animals treated with UA, a significant reduction of VEGF, NO, IL-1β (interleukin-1β) and IL-6 levels was observed in comparison to the non-treated and normal levels. TIMP-1 and IL-2 levels in serum of animals treated with UA were higher than control group and normal levels. Moreover, UA was observed to lower the TNF-α level in the mice’s serum. A significant difference was observed on the ninth day, compared to control animals’ levels, 142.3 ± 1.15 and 571.8 ± 8.0 µg/mL, respectively [97].

A study by Xiang et al. [67] showed that the treatment with UA and US597 resulted in a smaller number of metastatic pulmonary nodules in B16-F10/C57BL/6 mice than in the control group. Moreover, animals treated with UA and its prodrug survived for 12 weeks after the melanoma cell inoculation, in comparison to the control group (8 weeks). The treatment resulted in no significant loss in bodyweight and no remarkable changes in the tissues such as the heart, liver, spleen, kidney and small intestine. Blood tests showed a decrease in white blood cells and lymphocytes and slight changes in haemoglobin and platelet counts in the UA/US597 group; however, all results were within normal ranges. Immunohistochemical staining revealed a difference in the inhibition of the expression of ICAM-1 in the lung tissue of metastatic mice. The control group showed a marked enhancement of ICAM-1 expression in blood vessel endothelial cells. At a low US597 dose, no significant difference was seen, whereas the administration of UA and high doses of US597 resulted in a decrease of ICAM-1 expression.

Caunii et al. [66] used fertilized eggs, divided into two groups: a control and a test group which was inoculated with SK-MEL-2 tumour cells. All embryos showed similar survival and viability rates. OA and UA caused changes in the number of vessels inside the area of application in comparison to the control specimens. However, OA severely reduced the vascular density in a chosen area compared to the control and UA-treated samples. UA did not induce significant changes, nor inhibit melanoma growth. Despite antiangiogenic changes, neither OA nor UA caused the inhibition of the metastatic potential and inhibition of invasiveness of the SK-MEL-2 cells.

### 7.3. Ilexgenins

In a study by Yang at al. [36], 24 male C57BL/6J mice inoculated with B16-F10 cells, and were divided into four groups in a 10-day treatment with ilexgenin A (15 mg/kg), ilexgenin A (30 mg/kg),both through gavage, once a day, taxol (45 mg/kg) administered intraperitoneally twice a week and a control group which received 0.5% sodium carboxymethyl cellulose (CMC-Na) through gavage. Ilexgenin A significantly inhibited the growth of tumours in comparison to the control group; the weights of the tumours were, respectively, 4.87 ± 3.71 g and 10.01 ± 3.14 g. Comparing the effect caused by ilexgenin A and taxol, the results showed that taxol reduces the tumour growth almost twice as much as ilexgenin A, the average weights of the tumours were 2.88 ± 1.71 and 6.36 ± 3.99 g, respectively. Moreover, the ilexgenin A-treated group showed no metastases in the lungs, compared to the model group. Analysis of the tumour tissue revealed histopathological and morphological changes caused by ilexgenin A. In the group treated with 15 mg/kg of the compound atypical, usually epithelioid immature melanocytes with rapid mitotic activity were observed. Treatment with 30 mg/kg ilexgenin A resulted in large groups of melanocytes and single cells and small areas of necrosis. The taxol-treated group revealed large areas of focal necrosis, whereas the model group was observed to have large pieces of melanocytes and small areas of necrosis, similar to the ilexgenin A-treated group (30 mg/kg). Ilexgenin A was proven to be non-toxic to mice bearing the B16-F10 melanoma tumour; there were no significant differences between the treated animals and the control group in terms of symptoms and histological changes.

## 8. Limitations of the Studies Included in the Review

Regarding the in vitro studies, quite a significant number of reviewed experiments comprised the tests performed on murine cell lines, with only a slight predominance of the use of human melanoma cell lines. This might be a little surprising, as nowadays animal cell lines are only rarely used in cytotoxicity screening. Although most of the results cited indicated high or at least moderate antimelanoma activity of the examined compounds, still some studies reported the activity with IC_50_ values exceeding 100 or even 200 μM, which makes the compounds classified as inactive. The majority of the studies did not include the non-cancerous cells, as the reference for the safety or selectivity of the compounds tested. Likewise, no reference cytostatic was used in most of the studies, the use of which not only can enable the comparison of the potency of the examined compound, but also guarantees the proper course of the experiment. Generally, the results of the studies lack information on the prolonged exposure of the melanoma cells on the examined triterpenes, as most of them were incubated only for a standard 24 or 48 h. It would be interesting to observe the effect of the compounds in time, as some of the results clearly indicated an increase in their effectiveness. Moreover, the determination of the triterpenes’ impact on other aspects of melanoma cell functioning was only rarely described in the studies cited. Most of them focused only on cell viability, while other aspects, such as proliferation, cytoskeleton disruption or the synthesis of melanin, crucial for the effective elimination of cancer cells, were nearly ignored. Similarly, the mechanistic studies mainly described the effect of the compounds on apoptosis stimulation, or cell cycle arrest. Most of the studies lack more in-depth insight that would enable to preselect the compounds with multidirectional impact on melanoma cells. This, apart from the direct impact on the cell functions, should also include some aspects that often accompany along with the carcinogenesis process, i.e., inflammation, or excessive ROS production and cell damage. This problem should be studied more thoroughly in the future. Another issue that seems to be neglected is the concomitant treatment of triterpenes with the cytostatic drugs. Such treatment can sensitize cancer cells to cytostatic therapy, resulting in lowering the drug doses and mitigation of the side effect of such therapy. The published results so far, although scarce, indicated a synergistic effect of some of the triterpenes and, for example, taxanes, thus such experiments should be continued, also in further animal models.

As far as the animal studies are concerned, it should be underlined that, despite their promising results, they can only be regarded as preliminary observations. The main reason, apart from the scarce number of such studies, is the relatively small number of animals included, rarely exceeding 10 animals/experimental group. Moreover, in one or two studies, no number of the animals used was provided. Similarly, the results of the in vivo studies on the use of different strategies to improve the bioavailability of triterpenes are based only on the few experiments, performed within the small study groups. This cannot lead to drawing valid conclusions. Additionally, only a few in vivo studies focused on the side effects or toxicity of the examined triterpenes. Despite the fact that in most cases no toxic effect was noted, such approach should be included in the in vivo study, to provide information on the safety of the studied compounds. Interestingly, no in vivo studies exist for cucurbitacins, which should be an important direction for future studies, because it was one of the most promising groups, based on the results of the in vitro studies.

## 9. Conclusions and Perspectives

**Figure 15:**
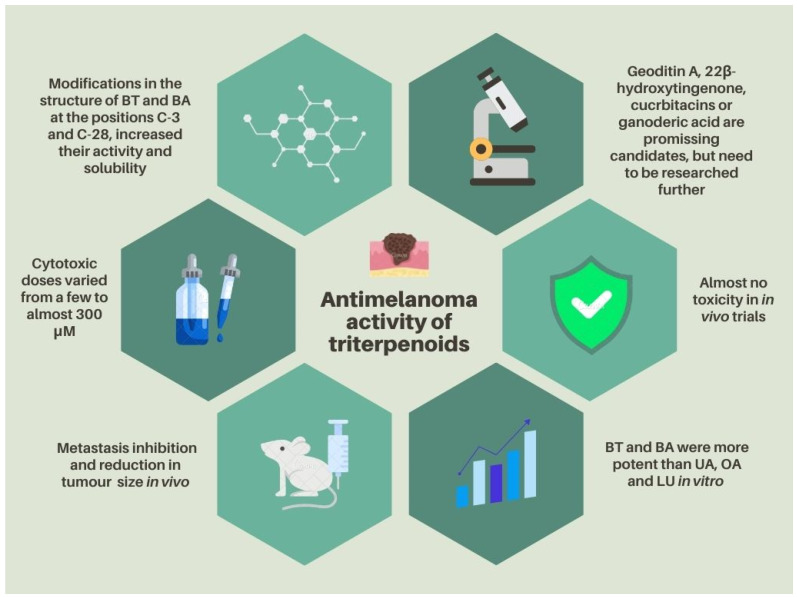
Graphical summary of the antimelanoma potential of natural triterpenoids.

Triterpenoids of plant origin may be considered as a valuable option for the development of effective antimelanoma agents (Figure 15).

Generally, triterpenoids display relatively low toxicity, an important aspect in the preselection of a candidate for a future drug. The available literature data indicate that BT, BA and their derivatives are more potent than LU, UA, OA and their derivatives. Cucurbitacins, which are less widely studied, seem to be a highly promising group of antimelanoma compounds. The results presented in Table 4 clearly show that the use of nanoparticles, ethosomes or complexation with cyclodextrins can significantly increase the antitumour activity of triterpenoids and increase their bioavailability. This is a well-justified line of further research that can provide new treatment options using compounds that are already available in nature. There is certainly much more to be done, especially with respect to compounds that are only briefly mentioned in this review, such as geoditin A, 22*β*-hydroxytingenone, cucurbitacins or ganoderic acid. Even though they have shown promising results, more research is required to obtain insight into their toxicity or mechanism of action, which remain unclear. The in vivo trials were run mostly on mice and their results indicated that triterpenoids could act not only as effective antiproliferative agents, as they reduced the size of induced tumours, but also as antiangiogenic agents. Synthesis of new derivatives based on structure–activity analysis is a promising strategy for further studies. Various modifications in the structure of both BT and BA, especially at the positions C-3 and C-28, increased their biological activity and solubility. This also means that the substituents at these positions are particularly important for the activity of both compounds. Therefore, further studies on the structure–activity relationships of these natural triterpenoids and on the mechanisms of action of their derivatives can bring future clinical benefits. Another interesting approach, for which the results of the so-far described experiments are very promising, is a wider use of drug delivery systems.

## Figures and Tables

**Table 3:** Mechanisms of cytotoxic activity of triterpenoid compounds and their derivatives.

Biological Effect and Cellular Mechanism	Compound	References
Apoptosis intrinsic pathway:		
cytochrome c release; mitochondria membrane depolymerization; caspase 3 and 9 activation; PARP-1 cleavage; MAPK cascade activation; Bcl-2, survivin downregulation; p53 upregulation; NF-κB inhibition; TNF*α* stimulation	Betulin, lupeol, betulinic acid, erythrodiol, oleanolic acid, ursolic acid, Cucurbitacins, masilinic acid, asiatic acid, poricoic acids, 25-hydroxy-3-epidehydrotumulosic acid, dehydroeburiconic acid, glycyrrhizic acid, boswellic acid, celastrol, ganoderic acid, 3-*O*-acetylursolic acid, taraxasterol	[3,4,7,25,30,32,45,46,51,56,64,66,68,73,80,81,82,83,84,85,86,87,88,89,90,91,92,93,94,95,96]
Autophagy:		
increase in Beclin-1 protein; activation of LC3 protein; depletion of autophagy-related gene 5	Ganoderic acid, ursolic acid	[33,80]
Inhibition of angiogenesis:		
inhibition of capillary formation and endthelial cells proliferation; upregulation of mTOR	Lupeol, ursolic acid, celastrol	[32,95,97]
Cell cycle arrest:		
cyclin D1, D2 downregulation; p21 upregulation; CDK2 inhibition	Odoratol, betulinic acid, betulin, lupeol, ursolic acid, oleanolic acid, maslinic acid, celastrol, ilexgenin A	[4,8,30,36,46,56,64,66,83,95]
Cytoskeleton disruption:		
cytoskeletal remodeling; attenuated stress fibre assembly; decrease in phospho-cofilin level; actin cytoskeleton disassembling by inhibition of Rho signaling	Lupeol	[57,98]
Inhibition of metastasis/migration:		
MITF downregulation via *β*-catenin and c-Raf-MEK1-ERK signaling pathways; decrease in production of VEGF, MMP-2, MMP-9 and NO modulating tumor adhesion and invasion steps by inhibition of focal adhesion signaling pathway including alterations in ICAM-1, VCAM-1, E-selectin, P-selectin, integrin *α*6*β*1, FAK, Src, paxillin and PTEN; inhibition of haptotaxis	Oleanolic acid, (23*R*, 24*E*)-acetoxymangiferonic acid, ursolic acid, lupeol, celastrol, betulinic acid, 22*β*-hydroxytingenone, 3-*O*-acetylursolic acid, taraxasterol	[64,67,68,71,95,97,98,99,100,101]
Inhibition of melanin production:		
downregulation of melanogenic proteins, aggravated with adenylate cyclase inhibitor SQ22536; increase in expression of MITF, a transcriptional factor of tyrosinase, Rab27a and myosin-Va; suppression of melanin accumulation; inhibition of melanogenesis by blockade of the mitogenic and differentiating signals from MAPK and Ras-MAPK kinase cascades; inhibition of *α*-melanocyte-stimulating hormone (MSH)	Geodotin A, lupeol, (23*R*, 24*E*)-acetoxymangiferonic acid, botulin, ursolic acid	[33,57,70,93,98,100]
Anti-inflammatory	Lupeol, betulin, betulinic acid, erythrodiol, oleanolic acid, ursolic acid, celastrol	[101,102]
Antioxidant		
increase in the activity of superoxide dismutase, glutathione S-transferase and glutathione peroxidase	Lupeol, betulin, betulinic acid, erythrodiol, oleanolic acid, ursolic acid, maslinic acid, celastrol	[76,77,95]

Abbreviations: mTOR—mammalian target of rapamycin kinase; MITF—melanocyte-inducing transcription factor; ERK—extracellular signal-regulated kinases; VEGF—vascular endothelial growth factor; MMP-2—matrix metalloproteinase-2; MMP-9—matrix metalloproteinase-9; ICAM-1—intercellular adhesion molecule 1; VCAM1—vascular cell adhesion molecule 1; FAK—focal adhesion kinase; Src—; PTEN—phosphatase and tensin homolog; MAPK—mitogen-activated protein kinase; Ras-MAPK—; MSH—melanocyte-stimulating hormone.

**Table 4:** Cytotoxic activity of triterpenoid compounds in various drug delivery systems.

Compound	Cell Line	Results	References
BT complexed with cyclodextrin	A-431^H^	Inhibition of proliferation ≈ 76% of control (C = no data)	[40]
BT silver nanoparticles	B164A5^A^	IC_50_ 0.9301 μM	[43]
	B16Ova^A^	IC_50_ 20.26 μM	
BT gold nanoparticles	RPMI-7951^H^	Cell viability: 75.1% (C = 25 μM) (24 h)	[111]
		Cell viability: 63.4% (C = 50 μM) (24 h)	
BT-ethosome formulation	B16-F10^A^	IC_50_ 2.43 μM (48 h)	[1]
PEGylated formulation of AgNP-B	B164A5^A^	IC_50_ 2.47 μM	[43]
	B16Ova^A^	IC_50_ 5.74 μM	
BA with cyclodextrin GCDG complex	B164A5 metastatic^A^	Cell viability: 42.33% (C = no data)	[51]
	B164A5 non-metastatic^A^	Cells viability: 50.3%	
BA-ethosomes formulation	B16F10^A^	IC_50_ 3.07 μM (48 h)	[1]
BA-HOBt loaded nanoparticles	A375^H^	Cell viability: 77.2% (C = 25 μM) (24 h)	[112]
		Cell viability: 69% (C = 50 μM) (24 h)	
OA-HOBt loaded nanoparticles	A375^H^	Cell viability: 81.2% (C = 25 μM) (24 h)	[112]
		Cell viability: 59.3% (C = 50 μM) (24 h)	
UA-HOBt loaded nanoparticles	A375^H^	Cell viability: 86.8% (C = 25 μM) (24 h)	[112]
		Cell viability: 74.8% (C = 50 μM) (24 h)	
CEL-NPs	B16-F10^A^	IC_50_ 2.81 μM (48 h)	[69]
Cuc-loaded L-MPs	B-16^A^	IC_50_ 464.37 μg/mL (48 h)	[73]
Cuc-loaded NPs	B-16^A^	IC_50_ 82.94 μg/mL (48 h)	[73]
Cuc-loaded S-MPs	B-16^A^	IC_50_ 283.41 μg/mL (48 h)	[73]

The letters in superscript next to the cell line name mean A—animal cell line, H—human cell line. Cell lines: A-431 human squamous carcinoma; B164A5, B-16, B16-F10 mouse melanoma; B16Ova murine melanoma transfected with an ovalbumin.

**Table 5:** Cytotoxic in vitro activity of natural triterpenoid derivatives against melanoma cell lines.

Compound	Cell line	Results	References
Betulin derivatives:			
28-*O*-propynoylbetulin	G361^H^	Cell growth: 10–32% of control (C = 10–20 μg/mL)	[80]
28-*O*-(3-butynyloxycarbonyl)betulin	G361^H^	Cell growth: 20–44% of control (C = 10–20 μg/mL)	
28-*O*-propargyloxycarbonylbetulin	G361^H^	Cell growth: 10–41% of control (C = 10–20 μg/mL)	
28-*O*-propynoylbetulin	C32^H^	Cell growth: 28.8% (C = 3 μg/mL); 3.6% of control (C = 10 μg/mL)	[84]
	A2058^H^	Cell growth: 45.9% (C = 3 μg/mL); 10.3% of control (C = 10 μg/mL)	
3-(2-propenoyl)betulin	Hs294T^H^	IC_50_ 69 μM	[39]
3-(3-butynyloxycarbonyl)betulin	Hs294T^H^	IC_50_ 164 μM	
3-propargyloxycarbonylbetulin	Hs294T^H^	IC_50_ 82.7 μM	
2-butynoyl derivative	Hs294T^H^	IC_50_ 10.6 μM	
Betulinic aldehyde derivative	Hs294T^H^	IC_50_ 74.4 μM	
Betulin-l-Ala-NH2	Me-45^H^	IC_50_ 86.6–10.1 μM (24–72 h)	[3]
Betulin-l-Dab-NH2	Me-45^H^	IC_50_ 66.5–9.3 μM (24–72 h)	
Betulin-l-Dap-NH2	Me-45^H^	IC_50_ 107.7–75.5 μM (24–72 h)	
Betulin-l-Lys-NH2	Me-45^H^	IC_50_ 55.3–2.5 μM (24–72 h)	
Betulin-l-Orn-NH2	Me-45^H^	IC_50_ 70.7–2.5 μM (24–72 h)	
Betulone	SK-MEL-2^H^	IC_50_ 3.3 μM	[9]
28-Hydroxy-(lup-20(29)-ene)-3-yl-2-(1*H*-indol-3-yl) acetate containing at the C-28 position a free hydroxyl group	A375^H^	Cell viability: ~70–65% (C = 83 and 167 µM)	[114]
	C32^H^	Cell viability: ~70–65% (C = 83 and 167 µM)	
30-Diethoxyphosphoryloxy-28-propynoylbetulin	C32^H^	IC_50_ 2.15 μM (72 h)	[44]
28-(2-Butynoyl)-30-diethoxyphosphoryloxybetulin	C32^H^	IC_50_ 0.76 μM (72 h)	
Betulinic acid derivatives:			
bet-CA (a DCA molecule has been appended on C-3 hydroxyl group of BA)	B16-F10^A^	IC_50_ 9.89 μM	[50]
BA + DCA	B16-F10^A^	IC_50_ 27.6 μM	
BA-TZ	RPMI-7951^H^	Cell viability: 54.7% (C = 10 μM)	[115]
		Cell viability: 24.5% (C = 50 μM)	
1*H*-Benzotriazole-1-yl (3*β*) 3-hydroxy-20(29)-lupaene-28-oate	A375^H^	Cell viability: 81.25% (C = 25 μM) (24 h)	[54]
		Cell viability: 69.8% (C = 50 μM) (24 h)	
4-isoquinolinyl amide of 3-*O*-acetyl-betulinic acid	A375^H^	EC_50 ¼_ 1.48 μM (72 h)	[116]
Lupeol derivatives:			
3*β*,28,30-lup-20(29)-ene triol	SK-MEL-2^H^	IC_50_ 14.5 µM	[9]
lupenone	SK-MEL-2^H^	IC_50_ 9.2 µM	
28,30-dihydroxy-3-oxolup-20(29)-ene	SK-MEL-2^H^	IC_50_ 10.8 µM	
Oleanolic acid derivatives:			
CDDO-Me (2-cyano-3,12-dioxo- oleana-1,9(11)-dien-28-acid methyl ester)	B16F10^A^	IC_50_ 5.85 µM	[117]
hederagenin	SK-MEL-2^H^	IC_50_ 13.8 µM	[9]
3-oxo-11*α*-methoxyolean-12-ene	SK-MEL-2^H^	IC_50_ > 30 µM (48 h)	[61]
3*β*-hydroxy-1-oxo-olean-12-en-28-oic acid	SK-MEL-2^H^	IC_50_ 11.2 µM (48 h)	
glut-5-en-3*β*-ol	SK-MEL-2^H^	IC_50_ > 30 µM (48 h)	
(3*S*)-*O*-(*N*-Methoxy-*N*-*a*-d-arabinosylglycyl) oleanolic acid	A375^H^	IC_50_ 9.6 µM (72 h)	[118]
(3*S*)-*O*-(*N*-Methoxy-*N*-*β*-l-xylosylglycyl) oleanolic acid	A375^H^	IC_50_ 9.7 µM (72 h)	
(3*S*)-*O*-(*N*-Methoxy-*N*-*β*-l-lyxosylglycyl) oleanolic acid	A375^H^	IC_50_ 8.4 µM (72 h)	
(3*S*)-*O*-(*N*-Methoxy-*N*-d-ribosylglycyl) oleanolic acid	A375^H^	IC_50_ 6 µM (72 h)	
9-[2-[[4-(3β-Acetyloxy-olean-12-en-28-oyl)-1-piperazinyl] carbonyl] phenyl]-3,6-bis(diethylamino]-xanthylium chloride (RhodOA)	375^H^	Cell viability: ~75% (C= 80 nM) (72 h)	[119]
		Cell viability: ~65% (C = 100 nM) (72 h)	
1*H*-Benzotriazole-1-yl (3*β*) 3-hydroxyolean-12-en-28-oate	375^H^	Cell viability: 87.4% (C = 25 μM) (24 h)	[54]
		Cell viability 62.5% (C = 50 μM) (24 h)	
Ursolic acid derivatives:			
3*α*, 6*α*, 30-trihydroxy-ursan-28-oic acid	B16F10^A^	IC_50_ 72.72 μM (48 h)	[120]
UA: HPgammaCD (ratio 1:2)	A375^H^	IC_50_ 31.38 μM (48 h)	[63]
	SK-MEL 2^H^	IC_50_ 9.26 μM (48 h)	
	B164A5^A^	IC_50_ 16.08 μM (48 h)	
UA:HPbCD (ratio 1:2)	A375^H^	IC_50_ 51.73 μM (48 h)	
	B164A5^A^	IC_50_ 40.84 μM (48 h)	
3*β*-acetoxy-urs-12-en-28-oic acid hexamethylenediamine (US597)	B16F10^A^	IC_50_ 8.57 μM (24 h)	[67]
Ursolic acid derivatives:			
3-*O*-*β*-acetoxy-ursolic acid	HTB-140^H^	IC_50_ 19.85 µg/mL (24 h)	[65]
		IC_50_ 8.75 µg/mL (48 h)	
	A375^H^	GI_50_ 32 μM	[64]
		IC_50_ 30.08 µg/mL (24 h)	[65]
		IC_50_ 25.92 µg/mL (48 h)	
	WM793^H^	IC_50_ 30.99 µg/mL (24 h)	
		IC_50_ 14.18 µg/mL (48 h)	
Ursolic aldehyde	HTB-140^H^	IC_50_ > 100 µg/mL (24 h)	
		IC_50_ 19.28 µg/mL (48 h)	
	A375^H^	IC_50_ > 100 µg/mL (24 h)	
		IC_50_ > 100 µg/mL (48 h)	
	WM793^H^	IC_50_ > 100 µg/mL (24 h)	
		IC_50_ > 100 µg/mL (48 h)	
3-O-*β*-acetoxy-19*α*-hydroxy-ursolic acid	HTB-140^H^	IC_50_ > 100 µg/mL (24 h)	
		IC_50_ > 100 µg/mL (48 h)	
	A375^H^	IC_50_ > 100 µg/mL (24 h)	
		IC_50_ > 100 µg/mL (48 h)	
	WM793^H^	IC_50_ > 100 µg/mL (24 h)	
		IC_50_ > 100 µg/mL (48 h)	
Uvaol	HTB-140^H^	IC_50_ > 100 µg/mL (24 h)	
		IC_50_ 93.62 µg/mL (48 h)	
	A375^H^	IC_50_ > 100 µg/mL (24 h)	
		IC_50_ > 100 µg/mL (48 h)	
	WM793^H^	IC_50_ > 100 µg/mL (24 h)	
		IC_50_ > 100 µg/mL (48 h)	
1*H*-Benzotriazole-1-yl (3*β*) 3-hydroxyurs-12-en-28-oate	A375^H^	Cell viability: 77% (C = 50 µM) (24 h)	[54]
Dammarane derivatives:			
3*β*-oleate-20*S*-hydroxydammar-24-en	B16F10^A^	IC_50_ 181.34 μg/mL (24 h)	[121]
20*S*,24*S*-epoxy-3*β*-oleate-25-hydroxydammarane	B16F10^A^	IC_50_ 98.4 μg/mL (24 h)	
20*S*-hydroxydammar-24-en-3-on	B16F10^A^	IC_50_ 22.95 μg/mL (24 h)	
3*β*,20*S*-dihydroxydammar-24-en	B16F10^A^	IC_50_ 49.57 μg/mL (24 h)	
20*S*,24*S*-epoxy-3*β*,25-dihydroxydammarane	B16F10^A^	IC_50_ 95.27 μg/mL (24 h)	
Glycyrrhetinic acid derivatives:			
NPC-402	B16F10^A^	IC_50_ 16 μM (24 h)	[122]
	A375^H^	IC_50_ 27 μM (24 h)	
	SK-MEL-28^H^	IC_50_ 33.5 μM (24 h)	

The letters in superscript at the cell line name mean A—animal cell line, H—human cell line. Cell lines: G-361 human Caucasian malignant melanoma; C-32 human amelanotic melanoma; A2058 human Caucasian metastatic melanoma; Hs294T human melanoma; SK-MEL-2 human malignant melanoma; Me-45 human malignant melanoma; B164A5 mouse melanoma; B16F10 murine melanoma; A375 human malignant melanoma; C32 amelanotic human melanoma; HTB-140 human melanoma cells derived from metastatic site; WM793 human stage I primary melanoma; 518A2 human melanoma; RPMI-7951 human malignant melanoma; SK-MEL-28 human malignant melanoma. Abbreviations: C—concentration; IC_50_—median inhibitory concentration.

**Table 6:** Examples of betulin, betulinic acid and lupeol derivatives.

Compound	Figure 2 Substituents	
	R_1_	R_2_
**1**	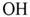	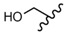
**2**	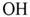	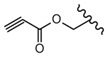
**3**	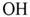	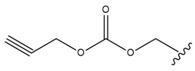
**4**	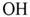	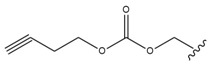
**5**	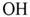	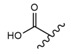
**6**	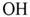	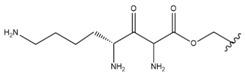
**7**	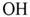	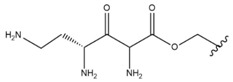
**8**	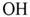	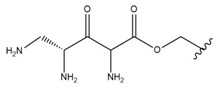
**9**	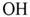	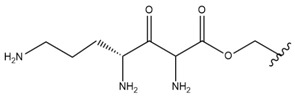
**10**	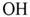	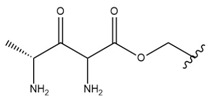
**11**	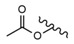	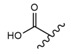
**12**	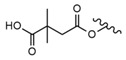	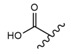
**13**	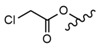	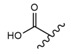
**14**	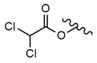	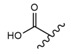
**15**	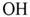	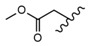
**16**	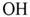	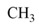
**17**	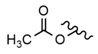	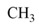
**18**	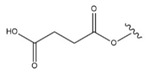	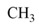
**19**	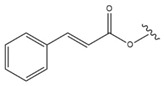	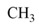
**20**	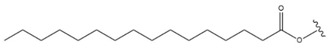	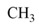
**21**	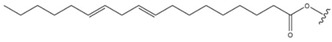	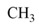
**22**	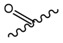	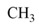
**23**	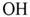	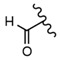
**24**	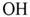	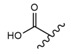
**25**	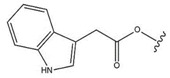	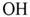

**Table 7:** Examples of betulin, betulinic acid and lupeol derivatives.

Compound	Figure 8 Substituents	
	R_1_	R_2_
**26**	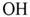	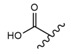
**27**	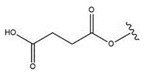	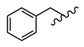
**28**	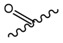	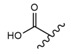
**29**	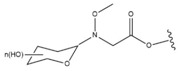	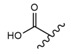
**30**	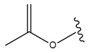	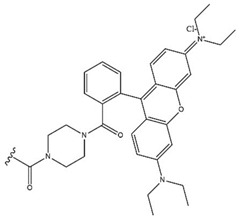

**Table 8:** Examples of betulin, betulinic acid and lupeol derivatives.

Compound	Figure 10 Substituents		
	R_1_	R_2_	R_3_
**31**	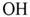	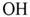	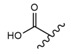
**32**	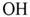	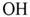	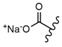
**33**	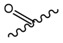	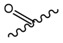	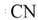
**34**	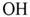	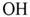	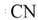
**35**	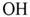	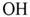	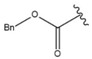
**36**	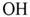	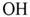	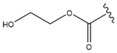

**Table 9:** Examples of celastrol derivatives.

Compound	Figure 12 Substituents
	R_1_
**37**	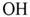
**38**	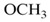
**39**	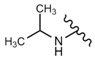
**40**	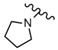
**41**	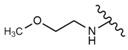
**42**	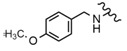
**43**	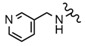
**44**	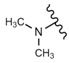
**45**	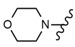
**46**	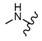
**47**	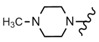
**48**	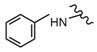
**49**	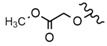
**50**	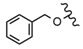
**51**	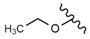
**52**	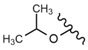

**Table 10:** Examples of ursolic acid derivatives.

Compound	Figure 13 Substituents	
	R_1_	R_2_
**53**	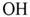	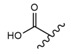
**54**	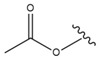	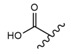
**55**	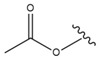	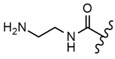
**56**	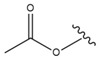	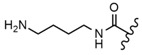
**57**	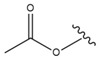	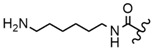
**58**	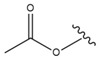	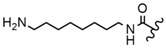
**59**	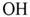	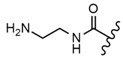
**60**	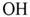	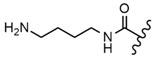
**61**	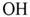	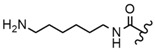
**62**	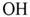	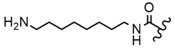

## Data Availability

Not applicable.
